# Supplementary figures and images for: Multi-omics analyses of Bacillus amyloliquefaciens treated mice infected with Schistosoma japonicum reveal dynamics change of intestinal microbiome and its associations with host metabolism
Source: PLoS Negl Trop Dis. 2024 Oct 28;18(10):e0012583. doi: 10.1371/journal.pntd.0012583 (PMC11515987; doi:10.1371/journal.pntd.0012583)

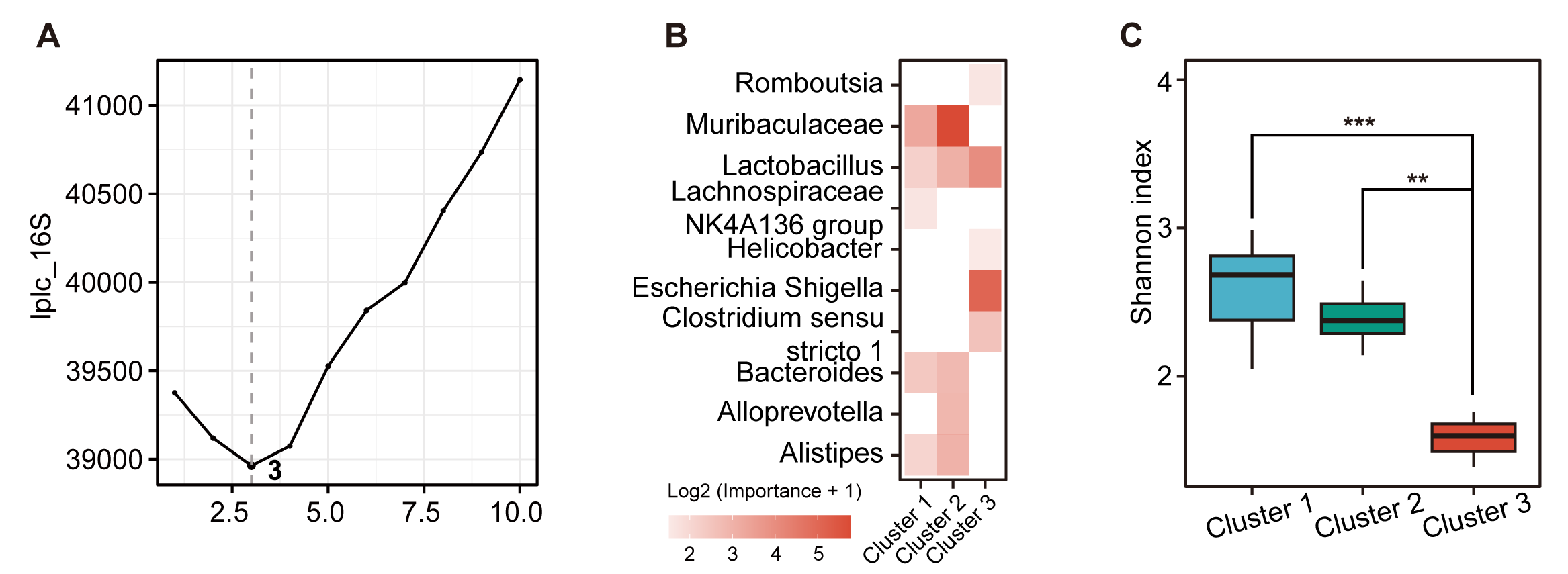

Supplement: S1 Fig — (A) The robustness of models evaluated by fitting curve. Grey dashed line represented the number of most stable DMM clusters. (B) Genera that contributed the most to the accuracy of the DMM for each cluster. The importance values were log-transformed. (C) Shannon index of each cluster. Data analysis was performed by Mann-Whitney U test. ** means P < 0.01, *** means P < 0.001. (TIF) [file pntd.0012583.s001.tif]

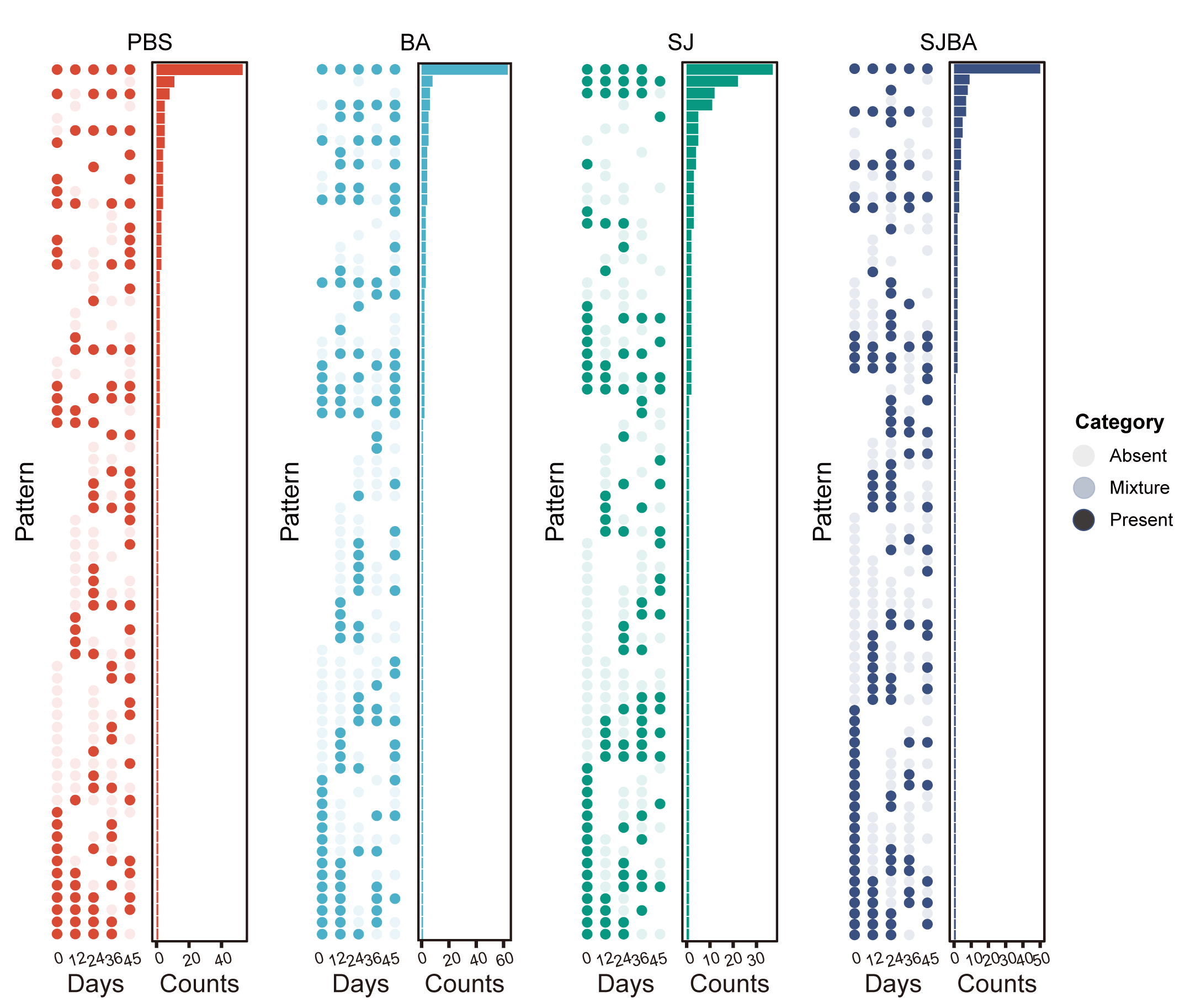

Supplement: S2 Fig — Genera at five time points were used to summarize the occurrence patterns in the four groups. Dark points represented the presence of bacterial genera (n = 3). White points represented the absence of bacterial genera (n = 0). Light points represented the transition between “presence” and “absence”. The length of the bar represented the counts of each pattern. (TIF) [file pntd.0012583.s002.tif]

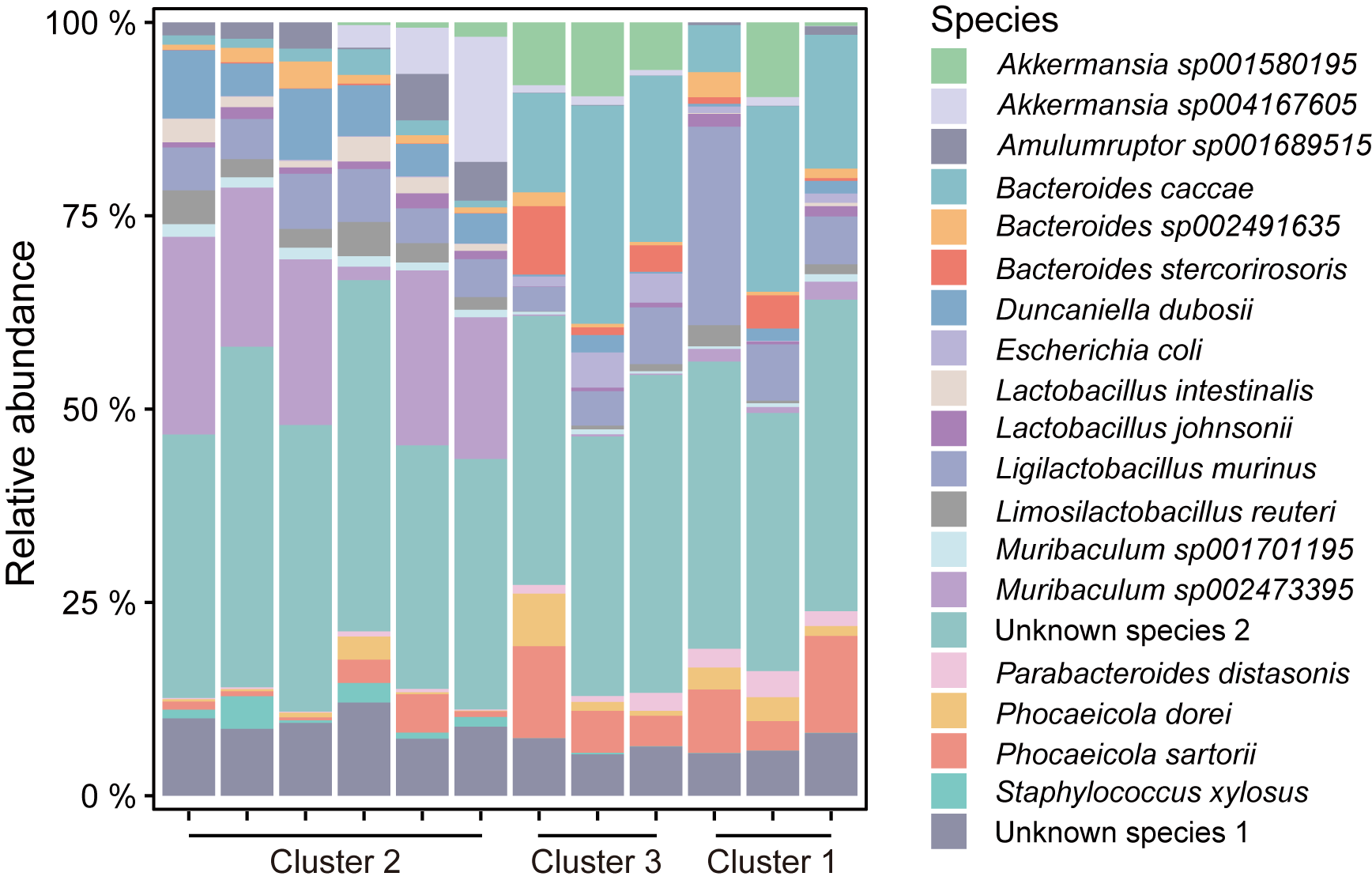

Supplement: S3 Fig — (TIF) [file pntd.0012583.s003.tif]

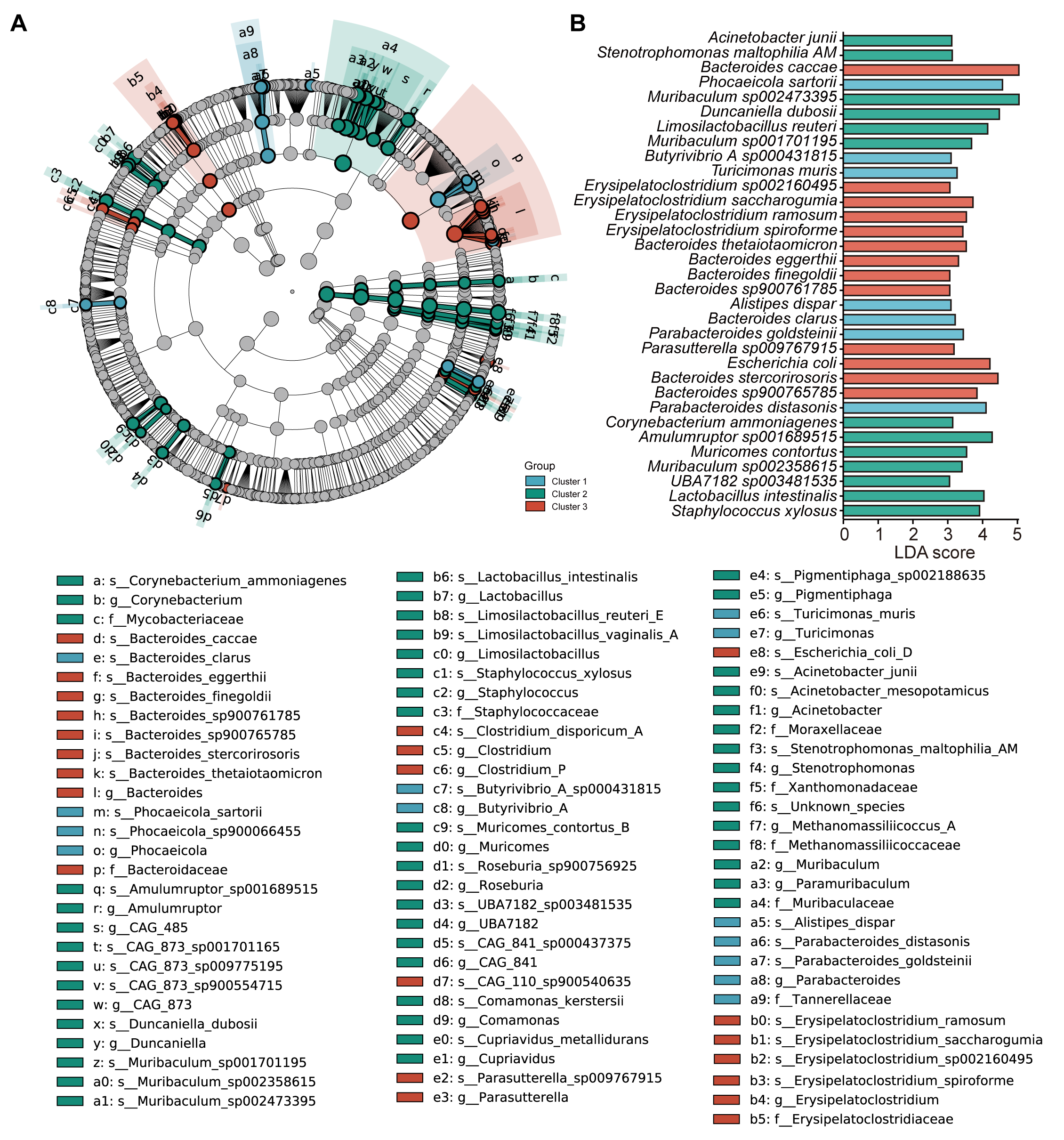

Supplement: S4 Fig — (A) A cladogram showing the discriminated taxa in different groups. (B) A histogram with LDA scores in four groups. Species highlighted in different colors indicate overrepresentation in the corresponding groups. The threshold of significance was set at 0.05. The threshold of the LDA score was set at 3.0. (TIF) [file pntd.0012583.s004.tif]

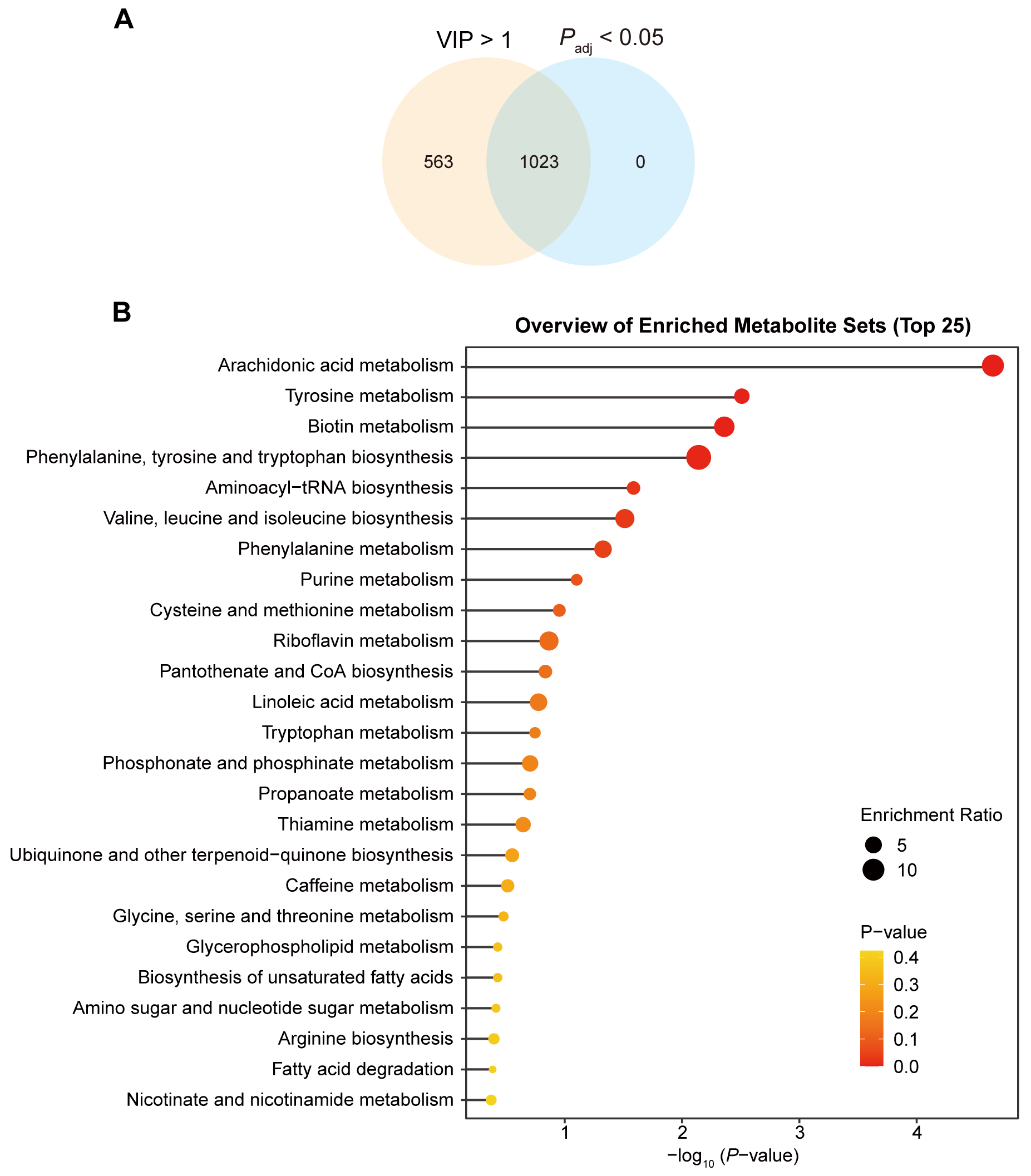

Supplement: S5 Fig — (A) Overlaps of screening of differential metabolites based on VIP value and adjusted P-value. VIP value was calculated by OPLS-DA analysis. P-value was analyzed by Mann-Whitney U test and adjusted by FDR. (B) Enrichment analysis of KEGG pathways based on differential metabolites. Size of the points represented the ration of metabolites enriched in KEGG pathways. The degree of color of points represented the degree of P-value. (TIF) [file pntd.0012583.s005.tif]

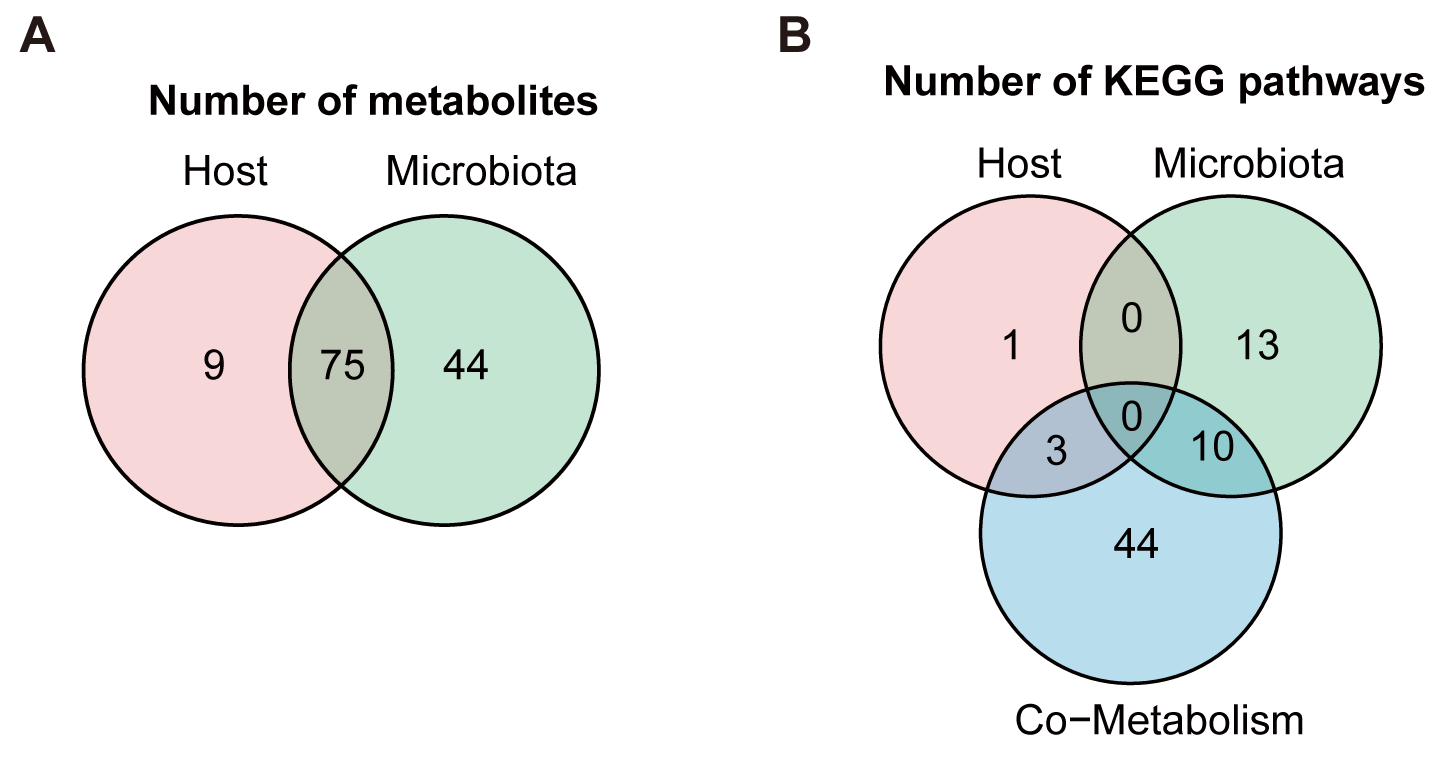

Supplement: S6 Fig — (A) Overlaps of metabolites sourced from host and microbiota. (B) Overlaps of KEGG pathways based on the metabolites sourced from host and microbiota. (TIF) [file pntd.0012583.s006.tif]

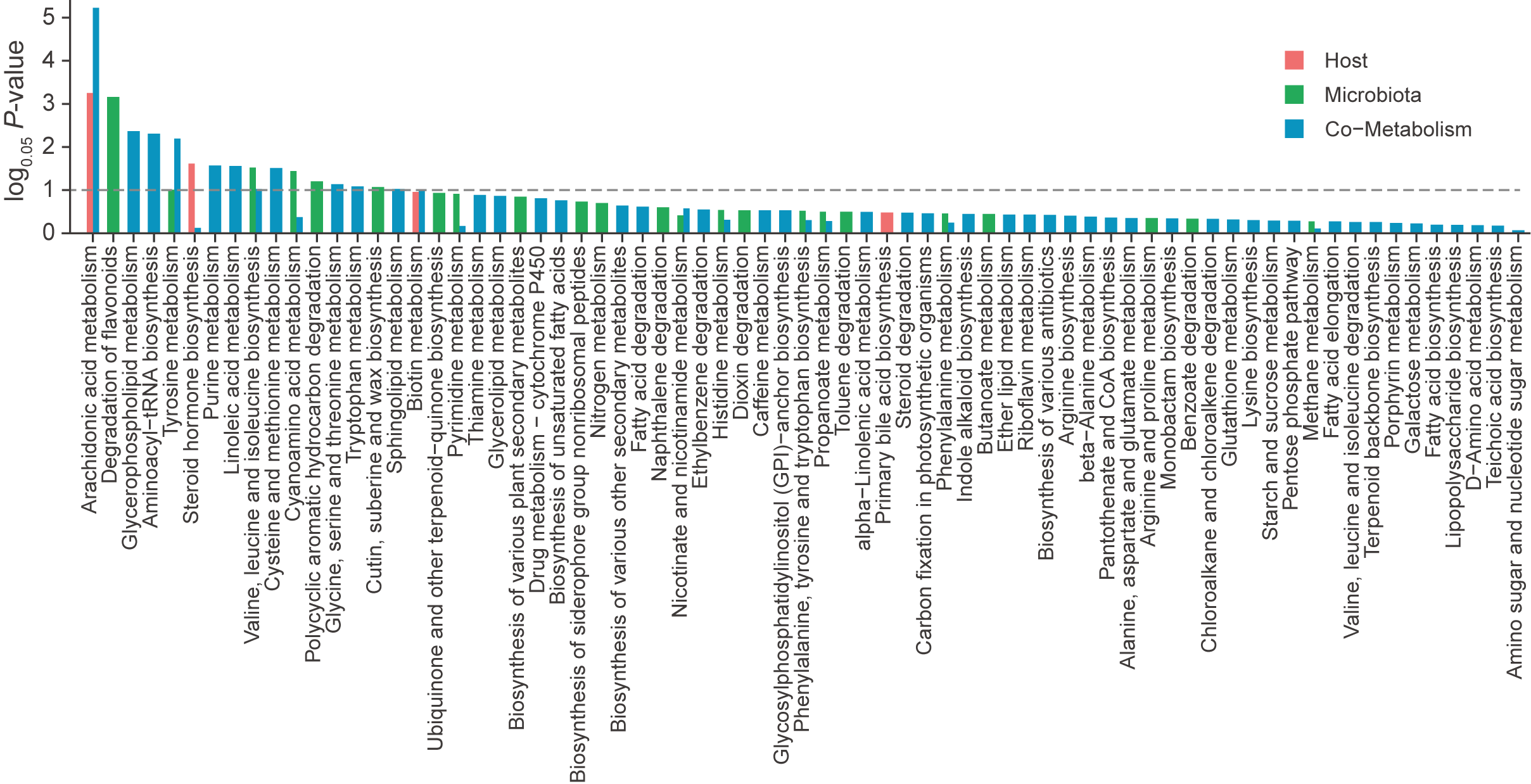

Supplement: S7 Fig — (TIF) [file pntd.0012583.s007.tif]

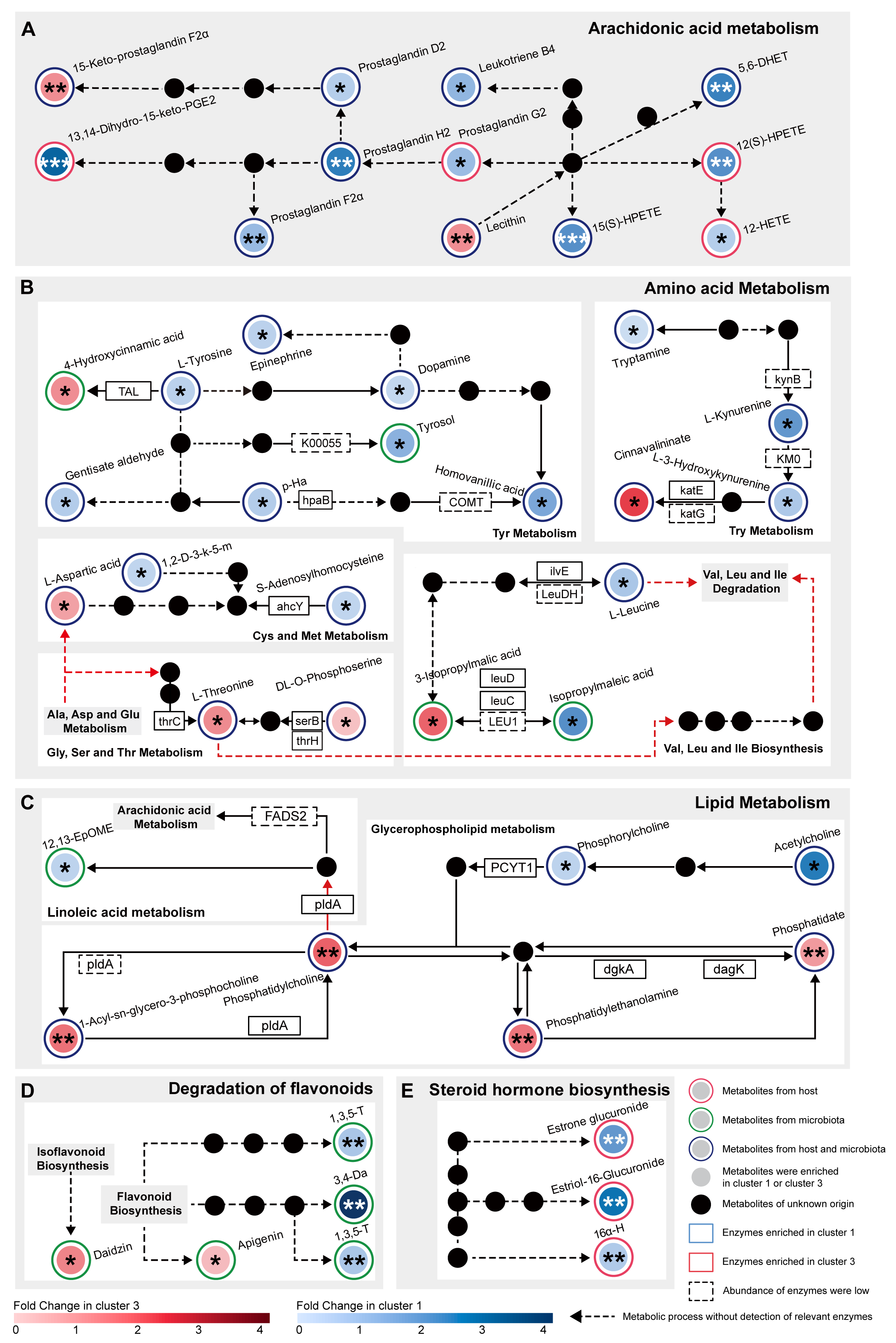

Supplement: S8 Fig — (A-E) Representative differential metabolites, relative enzyme-encoding genes, and involved metabolic pathways. The pathways were constructed based on the KEGG metabolic maps. Metabolites were indicated as red (enriched in the cluster 3 group), blue (enriched in the cluster 1 group), or black (none detected) balls. Identified microbial enzyme-encoding genes were represented in boxes (the dashed one means poor abundance). The dashed arrow indicated the potential metabolic process without detection of relevant enzyme-encoding genes. Different colors of circles outside the balls represented the sources of the metabolites. The degree of color of balls represented degree of fold change of metabolites. * means P < 0.05, ** means P < 0.01, *** means P < 0.001. Data analysis was performed by Mann-Whitney U test. (TIF) [file pntd.0012583.s008.tif]

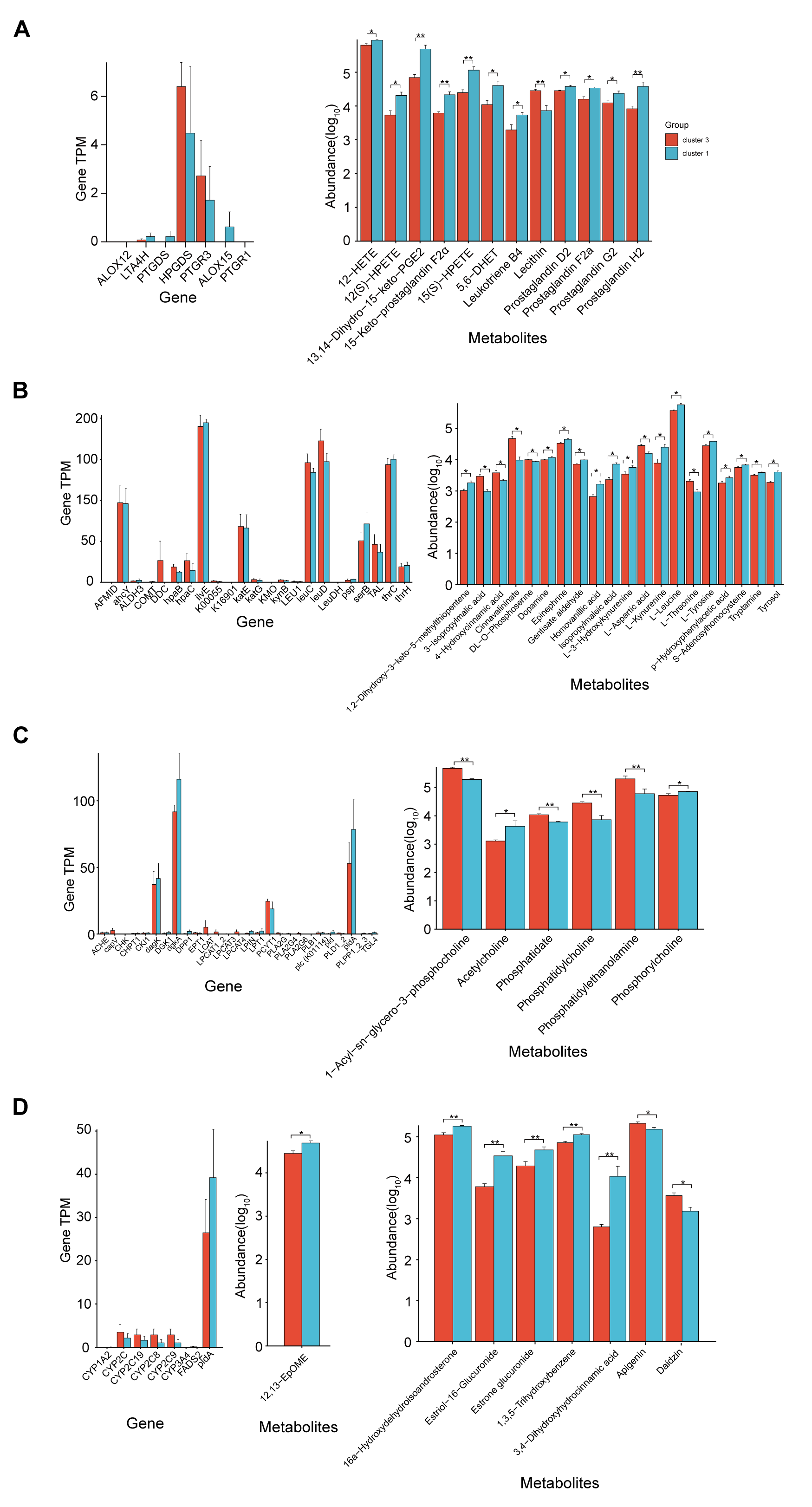

Supplement: S9 Fig — (A-D) Significance analysis of representative differential metabolites with exact sources and enzyme-encoding genes which involved into the KEGG metabolic pathways. * means P < 0.05, ** means P < 0.01, *** means P < 0.001. Data analysis was performed by Mann-Whitney U test. (TIF) [file pntd.0012583.s009.tif]

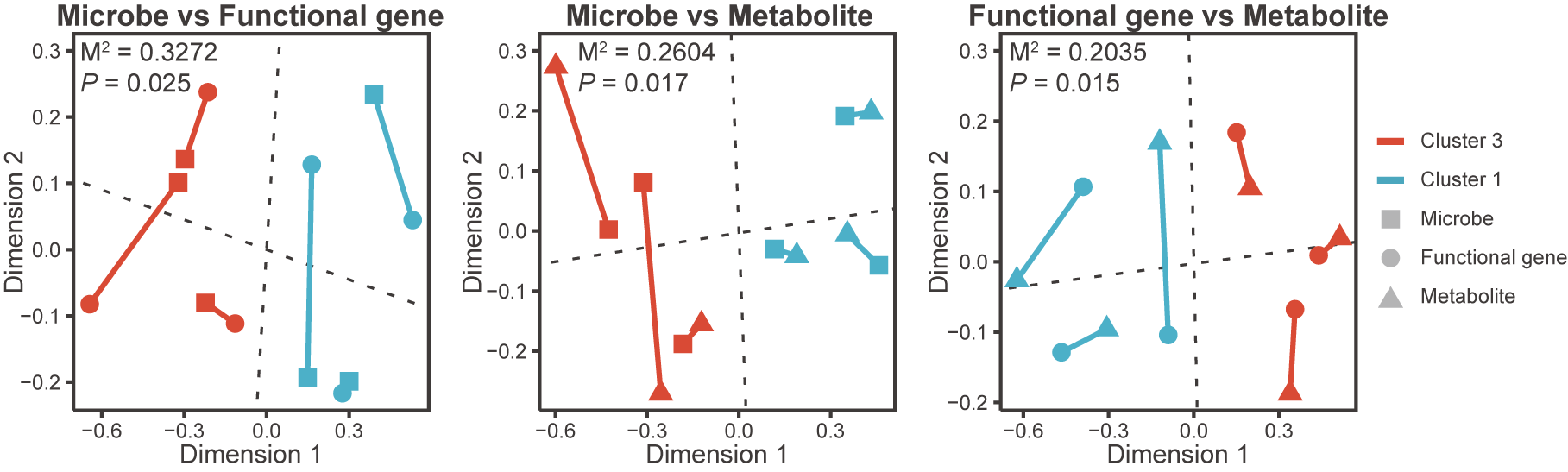

Supplement: S10 Fig — Different colored lines indicate different groups. Squares indicate differential intestinal microbiota, dots indicate differential functional genes, and triangles indicate differential metabolites. M2 represents the sum of squares of deviations. (TIF) [file pntd.0012583.s010.tif]

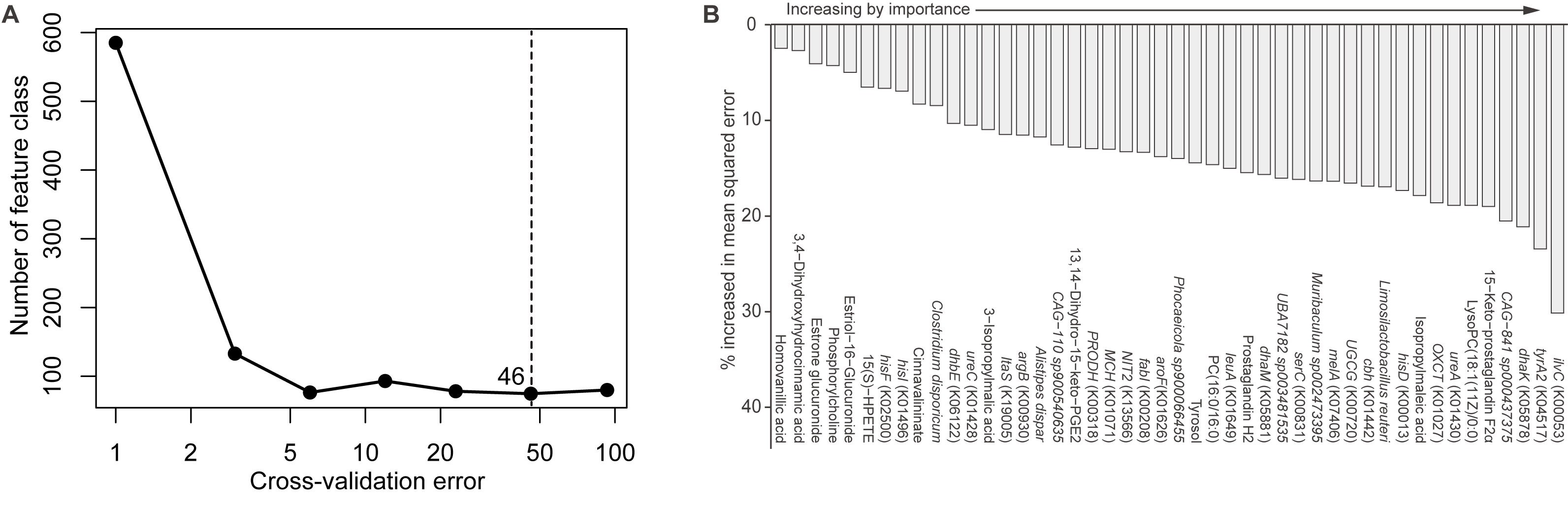

Supplement: S11 Fig — (A) Line point panel represented 10-fold cross-validation error as a function of the number of input classes used to regress against representative differential metabolites with exact sources and enzyme-encoding genes of two groups in order of variable importance. (B) Identification of representative intestinal microbiota, biofunctional genes, and serum metabolites by applying Random Forests regression of abundance. (TIF) [file pntd.0012583.s011.tif]

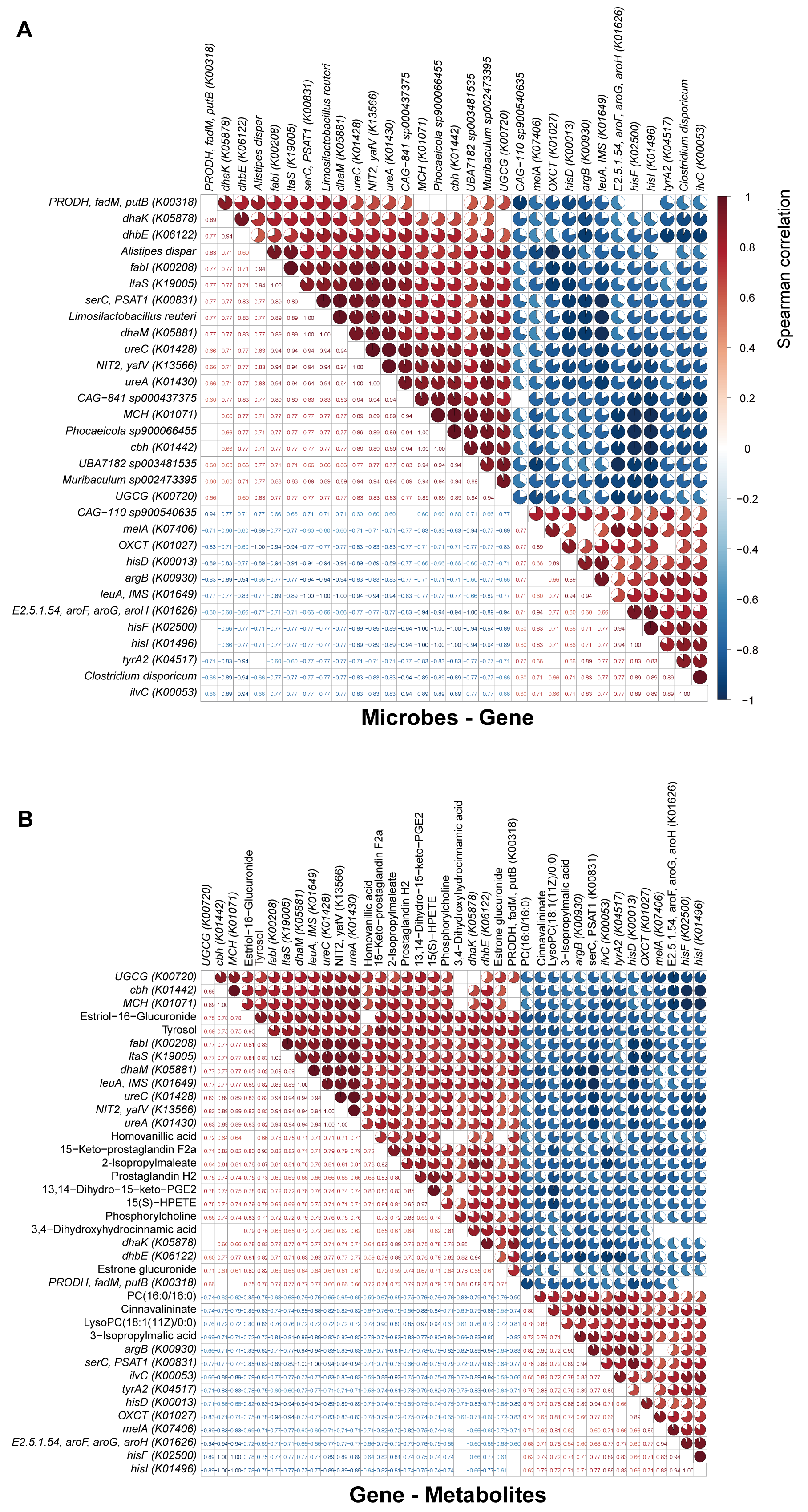

Supplement: S12 Fig — (A-B) Correlation between microbes with genes, and genes with metabolites. The completeness of circular pies represented the level of correlation. The threshold of P-value was 0.05, and was corrected by FDR. (TIF) [file pntd.0012583.s012.tif]
