# Supplementary material for: Multi-omics analyses of Bacillus amyloliquefaciens treated mice infected with Schistosoma japonicum reveal dynamics change of intestinal microbiome and its associations with host metabolism
Source: PLoS Negl Trop Dis. 2024 Oct 28;18(10):e0012583. doi: 10.1371/journal.pntd.0012583 (PMC11515987; doi:10.1371/journal.pntd.0012583)
Supplement: Attachment — Submitted filename: PNTD-D-24-00599_review.pdf [file pntd.0012583.s013.pdf]

The manuscript by Chen et al., titled “Multi-omics analyses of Bacillus amyloliquefaciens treated mice infected with Schistosoma japonicum reveal dynamic changes in the intestinal microbiome and its associations with host metabolism,” explores the role of Bacillus amyloliquefaciens in mitigating pathological injuries in mice infected with Schistosoma japonicum by modulating the intestinal microbiome and metabolome. This manuscript builds on a 2023 study by the same group, employing a more comprehensive multi-omics approach and extending the scope of analysis from microbial diversity to more detailed functional and metabolic descriptions. The authors demonstrate the potential of Bacillus amyloliquefaciens in modulating host metabolism, providing a potential new avenue for alleviating schistosomiasis symptoms and lesions through the administration of the bacterium as a probiotic. The study shows that treating mice with B. amyloliquefaciens prior to their infection with S. japonicum leads to alterations in the microbiome, metagenome, and metabolome, which are used to explain how this bacterium alleviates some symptoms of the parasitic infection. I found the findings interesting and consider this a strong continuation of the previous manuscript.

General suggestions:
Improve the readability of the manuscript by making sentences clearer and enhancing the overall phrasing. Many errors could have been caught with a thorough language proofreading. I have included several suggestions below to improve the manuscript, but I must emphasize that this is not an exhaustive list. The authors should make further improvements beyond my suggestions, potentially hiring professional proofreading services.

Discussion section suggestions:
I think that the Discussion section of the manuscript repeatedly presents the results and includes too many speculative propositions, making it difficult to follow. Additionally, it should cite figures when talking about the findings in this study. Overall, the section needs improvement to effectively communicate the contributions and implications of the study in a broader context.

Conclusion section suggestions:
The section repeatedly mentions the intervention of B. amyloliquefaciens, which could be streamlined for better readability. The conclusions are not clearly distinguished from the results and discussion, making it harder to discern the key takeaways. The conclusions could provide more insight into the implications and future directions of the research.

Line-specific suggestions:
Line 19. "the intestine"
Line 20: "Probiotic therapy has been shown to be effective"
Line 23: "unknown"
Line 26: "B. amyloliquefaciens was found to significantly"
Line 29: "glycerophospholipids"
Lines 29-30: "and were shown to be significantly positively correlated"
Line 42: "as a potential strategy to alleviate"
Line 44: Abbreviate Bacillus amyloliquefaciens to B. amyloliquefaciens from here to the end of the text.

Line 44: "schistosomiasis japonica through the mediation of the intestinal microbiome"
Line 46: "joint analysis of the gut microbiome and host serum"
Line 47: Please clarify what does “intervention” mean here?
Line 47: "intestinal microbiome and the secretion of metabolites derived from the intestinal microbiome"
Line 49: Rephrase for clarity: "improve lipid metabolism disorder and hormone secretion in mice"
Line 50: “This study highlights the crosstalk between the intestinal …”
Line 60: Consider clarifying "Schistosoma japonicum is a type of schistosomiasis" to "Schistosoma japonicum is a parasitic worm that causes schistosomiasis."
Lines 65-66: Improve this sentence for clarity: ”Pathological symptoms are the host response to the mechanism of autoimmune response.”
Line 74: I suggest rephrasing this sentence to read something like: "Gut microbiota is a diverse community of microorganisms that reside in the digestive tracts of humans and other animals."
Line 82: “aggravates”
Line 88: Improve this sentence for clarity: “Bacillus subtilis is the first microbe applied to schistosomiasis japonica.” What does this mean? During medical treatment in humans? In mice? Please clarify.
Line 95: Why are the authors using “excellent” here? In other words, can the author inform which positive probiotic qualities.
Lines 104-107: Improve this sentence for clarity: “Through the analysis of intestinal microbiota and serum metabolites of host, the effectiveness of B. amyloliquefaciens intervention was evaluated, and the mechanism of schistosomiasis japonica relief was elaborated from the perspective of microbiome-host interaction.”
Lines 111-120:

- Specify the bacterial strain used in the study, including a reference to the relevant genome or database.
- Provide the same information for the Schistosoma japonicum strain.
- Although previously reported, include a brief description of the culturing and growth conditions for both the bacterial and parasite strains.

Line 122-123:
It appears that the authors are extracting total blood via the retro-orbital plexus route, as collecting whole blood from eyeballs is not feasible. If this was the procedure used, it typically requires anesthesia in mice. Please clarify why anesthesia was not mentioned or used in this case.
Line 136: There is no mention of any barcodes before here. Describe the library design, including where these barcodes are located.
Line 159: May be change to “Metabolomic profiling”?
Line 185-186. Rephrase to make it more clear: “Heatmap presented the number of genus belonged to different occurrence patterns …”
Line 205. “and annotated to HMDB and KEGG.”
Line 219: I believe it is called “Random Forest”. Change it in the rest of the manuscript if you agree.

Line 227: “orange lines”. Additionally: The end of the sentence seems to be missing. To show what?
Lines 236-238: For clarity, rephrase the whole sentence starting with: “According to results …”
Line 238: “to detect the impact”
Line 238: S1 Fig is not a result from this study, but from their previous 2023 manuscript. For me this does not make sense to include it here as this manuscript can be cited.
Line 242-244: “to cluster 3” … “to cluster 2”. “Additionally, the microbial community of cluster 1 was distinct from that of cluster 3”
Line 249: Avoid using tremendously, instead use something like “was significantly altered”.
Line 256-259: For clarity, rephrase sentence starting with “Intersection of genera …”
Line 275: “in mice”
Line 302: “..., and so on (Fig 2E)”. I would suggest avoiding being vague here. You might want to highlight here which ones are important to this study and potentially why.
L321. “with B. amyloliquefaciens”
L331. "accounts for high proportions". Write it more clearly.
L332. “From the 494 HMDB metabolites, 226 were annotated in the KEGG database and were used”
L372. Improve the sentence “Metabolism of amino acids and lipids was found to be more significant …” by completing the metric that is being referenced. What is more significant?
L375. “higher”.
L390. “It is worth noting”
L401. Add punctuation to the sentence.
Line 415. “Boxplot presents”.
L423. belonging/that belong
Line 431: "enables it to endure the low pH of gastric juice" - should be rephrased to "enables it to withstand the low pH of gastric juice".
Line 435: "was gavage to mice infected with" - should be "was administered via gavage to mice infected with".
Line 438: "the niche of the intestinal microbiota can remain stable and undisturbed" - should be rephrased to "the niche of the intestinal microbiota remains stable and undisturbed".
Line 442: "has always maintained state of dynamic balance" - should be rephrased to "has always maintained a state of dynamic balance".
Line 447: "one of the effectiveness of B. amyloliquefaciens intervention" - should be "one of the effects of B. amyloliquefaciens intervention".
Line 450: "which can only be the death and disappearance as final result" - should be rephrased to "resulting in the death and disappearance of other core bacterial genera".
Line 451: "the influence of Escherichia Shigella" - should be "the impact of Escherichia Shigella".
Line 454: "reached another relatively stable equilibrium state" - should be "reached a different but relatively stable equilibrium state".
Line 456: "be used as carbon source" - should be "be used as a carbon source".
Line 458: "to kill E. coli" - should be "to kill Escherichia coli".
Line 459: "But the specific mechanism still" - should be "However, the specific mechanism still".
Line 440: I would specify where this statement comes from "... composition and abundance of intestinal microbiota fluctuated during the experiment X, the core microbiota containing key species remained stable (Fig X)."
Line 443. I would rephrase "the immense change of intestinal microbiota was observed to be inhibited after intervention of B. amyloliquefaciens, so that the ecology of intestinal microbiota

remained stable in another state." Instead, I would change it to something similar to "the observed change in intestinal microbiota was inhibited following the intervention of B. amyloliquefaciens, as well as stabilizing the ecology of the intestinal microbiota in another state."
Line 435: Change this sentence as it is misleading: “B. amyloliquefaciens was gavage to mice infected with S. japonicum.”. Describe the experiment detailed in Methods, as B. amyloliquefaciens was gavage to mice one week before the infection with S. japonicum..
Line 459: This sentence is speculative. It should not be a fact stated but rather a plausible explanation: Something like “Therefore, B. amyloliquefaciens could be alleviating the dysbiosis of microbiota and remodeled the homeostasis of gut by regulating the competition of microbiota in the niche and its nutrient composition.”
Line 476-479: Clarify in which model it confers protection.
Line 480: Genus in italics.
Line 527: “play”
Lines 577-578: Follow Line 435 suggestion.
Lines 588-589: “In this study, we found that various metabolic pathways (steroid hormone biosynthesis, arachidonic acid metabolism, glycerophospholipid metabolism) are carried out in the liver.” Where have the authors demonstrated this?

Figure suggestions:
Fig 3. I suggest writing a more meaningful figure title, for example, instead of just mentioning cluster 1 and cluster 3, referencing the treatment that gives rise to these clusters. Panel (C) has too many details to be clearly seen for its size. I would suggest making a bigger plot and move it as a supplementary figure or reconfigure Fig 3, reducing panel (D) and maybe trimming the plot on (E). (E) Revise the phrasing for clarity.

Figure 4. Types are too small. I suggest breaking it up into two or more figures. Panel A, explain where the data comes from.

Figure 2. I suggest eliminating the dots color code and change it to cluster number next to the species (blue and turquoise are too similar, and red gets confused with abundance heatmap dots. Otherwise, picking different colors. Additionally, moving Kegg footnote reference to fit panel (E).

Figure 5. This figure has many elements that cannot be read clearly, such as barely visible numbers on the bar plots. It is complex and not self-explanatory, and the figure legend does not provide sufficient guidance to the reader. My suggestion is to make it clearer in every sense possible.
