# Supplementary material for: Multi-omics analyses of Bacillus amyloliquefaciens treated mice infected with Schistosoma japonicum reveal dynamics change of intestinal microbiome and its associations with host metabolism
Source: PLoS Negl Trop Dis. 2024 Oct 28;18(10):e0012583. doi: 10.1371/journal.pntd.0012583 (PMC11515987; doi:10.1371/journal.pntd.0012583)
Supplement: Attachment — Submitted filename: Review_PNTD-D-24-00599.doc [file pntd.0012583.s014.doc]

In this manuscript Chen et al describe the probiotic role of Bacillus amyloliquefaciens in dysregulated gut microbiota caused by Schistosoma japonicum. This investigation will provide a new strategy for adjuvant therapies of S. japonicum infection. The authors use an impressive variety of methods to test Multi-omics changes by administration of B. amyloliquefaciens and present a large amount of data that support probiotic potential of B. amyloliquefaciens. The presented data are quite standard for the field. The manuscript is very well written and, with one exception that is listed below, the conclusions are supported by the data shown. Given that Bacillus as combination therapy have already reported and the presented results are primarily bioinfomatic analyses. Despite observing some probiotic microbiota and metabolites are upregulated, lacking experimental evidence to confirm differential gut flora and metabolites caused by Bacillus amyloliquefaciens are relevant to the improvement of disease. Therefore, some experiments are advised be necessary for this paper as follow.

Major Issues:

1) Authours have previously published the alteration of gut microbiota in intestine of mice infected with Schistosoma japonicum after treatment with Bacillus amyloliquefaciens. What is difference compared to current investigation, data shown whether have overlapped?
2) Data provided by authors showed that B *. amyloliquefaciens* promote the growth of beneficial bacteria and inhibit potential pathogenic bacteria. In vitro inhibition assay should be performed to explore the possibility of B *. amyloliquefaciens* directly inhibit pathogen *Escherichia Shigella*. Although B. amyloliquefaciens induced autophagy play a role in killing bacteria, secretory antimicrobials are often more efficient to kill bacteria residing in the same niche. And increased beneficial bacteria after with intervention of B. amyloliquefaciens also should be discussed about its antibacterial effect or association with decreasing pathogenic bacteria. If authors have these probiotic isolates, I encourage you perform an in vitro killing bacteria assay to confirm in vivo results.
3) Authors detected differential metabolites, whether these metabolites are responsible for limiting of pathogens (such as *Escherichia Shigella* ) in vivo. Authors should picked out several metabolites to test antibacterial ability in vitro. If it would be hard to obtain these metabolites, related antibacterial investigations should be included in discussion.
4) I can not acquire colonization information about B *. amyloliquefaciens* from this paper, does it can colonize in intestine of mice and how long persist？If it can not survive or colonize in vivo well, whether non-living cells Bio-adjuvant would produce similar changes as observed after administration of living cells, if so, non-living cells may be safety.

5) IFN-γ, IL-12, TNF-α, and IL-4, IL-5, IL-13, TGF-β were shown to increase during infection, authors should measure these cytokines whether have changes after administration of Bacillus amyloliquefaciens, which should be considered for its application value in clinical treatment.

Minor Issues:

I suggest following changes in writing:

Line 47 dysbiosis should be replaced by restoration.

Line 102 “which can target and reshape the intestinal microbiota and has the potential to alleviate schistosomiasis japonica” should be edited as “which has the potential to alleviate schistosomiasis japonica by targeting and reshaping the intestinal microbiota” because previous sentence have mentioned the role of *B. amyloliquefaciensa* regulating gut microbiota.

.
