# Supplementary material for: Multi-omics analyses of Bacillus amyloliquefaciens treated mice infected with Schistosoma japonicum reveal dynamics change of intestinal microbiome and its associations with host metabolism
Source: PLoS Negl Trop Dis. 2024 Oct 28;18(10):e0012583. doi: 10.1371/journal.pntd.0012583 (PMC11515987; doi:10.1371/journal.pntd.0012583)
Supplement: Attachment — Submitted filename: Reply to reviewers.docx [file pntd.0012583.s015.docx]

**Response to Reviewer 1**

Dear reviewer, thanks for your patient review and detailed comments on our manuscript. We sincerely believe that your comments will be of great help in improving the quality of our manuscript. Based on your comments, we have made detailed modifications to our manuscript and added detailed description about methods. Besides, we have added an experimental design diagram to illustrate our experimental arrangement and added it to Figure 1. I hope the revised manuscript will meet your expectation. Our modifications and answers to your questions points to points were presented below.

**Parts of Methods**

The study design was appropriate and addressed the objectives, however, authors need to explain the following for clarity:

Line 103: Can authors please explain intragastrical administration in more detail? Is this directly administered in the stomach via needle or oral gavage?

**Reply**: Thanks for pointing out this problem. Actually, we conducted intragastrical administration via oral gavage. We have supplemented it based on your suggestion and improved the description about experimental details in part of methods. Please refer to line 104: “intragastrical administration via oral gavage of B. amyloliquefaciens”, and line 130-131: “Intragastric administration via oral gavage of PBS and *B. amyloliquefaciens*”.

Line 110: Why are only female mice? What was their diet? Where did authors purchase S. japonicum? Or did authors culture it?

**Reply**: Thanks for your questions. In the experimental design period, we referred to the methods of selection of mice in this article (doi: 10.3389/fimmu.2020.569727). Meanwhile, there is currently no research reporting the impact of gender on schistosomiasis japonica. That was why we chose female mice. All the mice were under standard rat food, and we have improved the detail about the feeding of mice in the methods, please refer to line 122-124: “All mice were settled in plastic cages with metal fence covers. The environmental conditions were sterile, with free access to standard rat food and drinking water under controlled temperature (25 ± 5°C), humidity (60%–70%), and light (12/12-hour light/dark cycle).” *S. japonicum* was cultured in our lab and we have also supplemented the detail about it, please refer to line 117-119: “Cercariae came from the Chinese mainland strain of *S. japonicum* which was maintained by serial passage through *O. hupensis* snails and the inbred Chinese Kun-ming mice”.

Line 117: How long did it take for cercariae to penetrate skin?

**Reply**: According to the textbook, it only takes a maximum of 20 seconds for the cercaria to enter the body from contact with the host's skin. To ensure the success of the model construction, we kept the process of attaching the cover glass with cercaria to the shaved abdominal skin for 30 minutes, allowing the cercaria to fully penetrate into the host's body. We also have added this detail into methods, please refer to line 129-130: “Cover slips containing 27 ± 3 S. japonicum cercariae were covered on the shaved abdominal skin of mice for 30 minutes to ensure complete penetration of the cercariae into the skin.”.

Line 118: What amount of B. amyloliquefaciens was administered? Concentration? It would be beneficial to make a figure outlining time and administration of treatments.

**Reply**: Thanks for your suggestion. The using concentration of *B. amyloliquefaciens* was 1×10^9^ CFU/mL. We have supplemented the detailed information on line 131. And based on your suggestion, we have made a figure about our experimental design, please refer to the figure below and **Fig 1A** in the manuscript.

Line 124: How long was the entire study? How long was the treatment?

**Reply**: The entire study lasted for 52 days, from adaptive feeding period (seven days) to the day 45 after infection of *S. japonicum*. And the treatment of *B. amyloliquefaciens* lasted the whole entire study for 52 days. Detailed information was described on line 131-133: “Intragastric administration via oral gavage of PBS and *B. amyloliquefaciens* (1×10^9^ CFU/mL) was conducted every three days, starting from one week before the infection till the day of mice sacrifice. The dose of each intragastric administration was 0.3 mL. All mice were sacrificed on day 45.” You can also refer to the figure above and **Fig 1A** in the manuscript for detailed information about the experiment design.

Line 126: Please explain reasoning for timepoints.

**Reply**: Thanks for pointing out this problem. “Five sampling time points” were day 0, day 12, day 24, day 36 and day 45 after infection of *S. japonicum*, respectively. We wanted to observe the dynamic changes of gut microbiota over time, and 12 days as an interval is neither long nor short, which was very suitable. The reason why day 45 infection of *S. japonicum* were selected to be one sampling time point was that because the model mice were on the brink of death due to worsening symptoms of schistosomiasis on day 45, so we had to collect samples and sacrifice mice. That was the reason why we chose five sampling time points. We have improved the description of it, please refer to line 143-144: “Three stool samples from each of the four groups at five sampling time points (day 0, day 12, day 24, day 36, day 45 after infection of *S. japonicum*) were selected for 16S rRNA gene sequencing (**Fig 1A**).”.

Line 126: How were stool samples collected?

**Reply**: We used the “stress defecation” method to collect stool from mice. And we have supplemented the detailed description about it on line 136-137: “Specifically, the anus of mice was stimulated with cotton swabs to promote defecation, and then collected fresh stool in sterile tubes.”.

Line 160: Why wasn’t the PBS control group examined?

**Reply**: Thanks for raising this question. To be horst, we just wanted to investigate the changes of serum metabolites before and after treatment of *B. amyloliquefaciens*, and we didn’t pay much attention to the changes of serum metabolites in mice of PBS group. Furthermore, the information of changes of serum metabolites in mice of PBS group wasn’t very useful to subsequent analysis based on our experimental design. To avoid redundancy and waste of data, we just picked samples from SJ and SJBA groups for subsequent analysis.

**Parts of Results**

The results were presented clearly and match the analysis. Authors did a great job putting the figures together and explaining the results section.

**Reply**: Thanks for your agreement.

**Parts of Conclusions**

Discussion and conclusion were very detailed and well written. Authors explained how the data can be helpful in advancing the use of probiotics like, B. amyloliquefaciens as a treatment for infections.

**Reply**: Thanks for your agreement.

**Parts of Editorial and Data Presentation Modifications**

Minor revisions.

**Reply**: Thanks for your suggestion. We have checked content and expression of manuscript carefully.

**Parts of Summary and General Comments**

Please have authors address the following:

1. Please be mindful of grammar and punctuation.

**Reply**: Thank you for your suggestion. We have carefully checked the grammar and punctuation, and corrected any errors.

2. What changes in host metabolism occur during schistosoma japonicum infection?

**Reply**: Thanks for raising this problem. Suggested by the results, some metabolites were significantly enriched such as L-threonine and phosphoserine from glycine, serine and threonine metabolism, phosphatidylcholine and phosphatidate from glycerophospholipid metabolism (refer to figures below and Fig S9 in the manuscript, red meant the significant enrichment of metabolite after infection of *Schistosoma* *japonicum*). And these results were the same as others research (doi: 10.3389/fimmu.2020.569727, 10.1371/journal.pntd.000080), like “reduced the levels of tricarboxylic acid cycle intermediates, increased the levels of amino acids, choline, and urinary 3-ureidopropionate, and perturbed lipid metabolism, glycolysis stimulation, tricarboxylic acid cycle, and a series of microbial-related metabolites”.

3. Line 40: Is this the only known drug on the market for this parasitic infection?

**Reply**: According to some references, praziquantel is the most effective and widely used compound on treatment of schistosomiasis, and it is the current therapeutic drug of choice for treating patients diagnosed with schistosomiasis (doi: 10.1038/s41572-018-0013-8). But it’s not the only known drug.

4. Line 42: What are the intestinal impacts of schistosome infections?

**Reply**: Schistosomiasis japonica can lead to intestinal granulomas accompanied by inflammation, necrosis, and rupture of intestinal mucosal tissue. In the middle and late stages of schistosomiasis, granulomas develop into fibrosis. We have added the description about it into manuscript. Please refer to line 43: “granuloma and fibrosis induced by schistosomiasis japonica in intestine”.

5. Line 43: What are the clinical symptoms of this infection?

**Reply**: Thanks for your suggestion. Schistosomiasis japonica can cause granulomas and fibrosis in the intestines and liver clinically. We have added the description about it into manuscript. Please refer to line 43: “granuloma and fibrosis induced by schistosomiasis japonica in intestine and liver”.

6. Line 51: Can authors elaborate on the “crosstalk” between host and microbiome?

**Reply**: Yes. The gut microbiota is a large group of microorganisms that reside in the human digestive tract, and their activities are closely related to human health. For example, the gut microbiota can break down some food that the human body cannot digest, producing beneficial substances (like short chain fatty acids) and enhancing human intestinal health. On the contrary, human physiological activities can also affect the composition and metabolic activity of the gut microbiome. The gut microbiota of people with diseases can also change, leading to an increase in the abundance of opportunistic pathogens and promoting the deterioration of the disease. Therefore, host-microbiome crosstalk is crucial and plays an important role in human health. And we also explained this relationship in our manuscript. Please refer to line 503-507: “Metabolites of intestinal microbiota are the bridge and important factor in microbiota-host crosstalk. These metabolites act as signaling molecules in the interaction between the host and microbiota and play a key role in guiding the physiological metabolism of the host. The intervention of probiotics can regulate the secretion of these metabolites, and ultimately improve the host health and prevent diseases through two-way communication between the gut and other organs”.

7. Line 63: Where is the fibrosis occurring?

**Reply**: Fibrosis always occurs in liver and gut of host. We have added this detail into manuscript, please refer to line 63: “fibrosis in liver and gut”.

8. Line 88: Species needs to be italicized.

**Reply**: Thanks for pointing out this problem. We have revised it.

9. Line 160: Why wasn’t the PBS control group examined?

**Reply**: Thanks for raising this question. To be horst, we just wanted to investigate the changes of serum metabolites before and after treatment of *B. amyloliquefaciens*, and we didn’t pay much attention to the changes of serum metabolites in mice of PBS group. Furthermore, the information of changes of serum metabolites in mice of PBS group wasn’t very useful to subsequent analysis based on our experimental design. To avoid redundancy and waste of data, we just picked samples from SJ and SJBA groups for subsequent analysis.

10. Line 282: Can authors please explain the n=0? Is this a mistake?

**Reply**: Yes, I can explain it and I confirm that is not mistake. According to method of the manuscript (line 213-216: “According to the occurrence frequency of genus at five time points in each of the four groups, three categories could be divided, “absent”, “mixture”, “present”. “Present” represented that genus occurred in all three samples in each of the four groups at five time points. “Mixture” represented that genus occurred in one or two samples, and “absent” means that genus didn’t occur in any samples.”), we have defined three categories about the occurrence frequency of genus. “n=0” meant that genus didn’t occur in any samples at five time points which showed as “Absent” with grey point in the **Fig 1D**, so the number was zero. Please refer to figure below and **Fig 1D** in the manuscript.

11. Line 468: Do authors plan on examining the expression any inflammatory cytokines?

**Reply**: Yes, we have such experimental plans. We plan to create a new batch of *Schistosoma japonicum* infection models and detect the expression of inflammatory cytokines such as IL-10, IL-13, IL-17, IFN-γ, NF-κB, TNF-α, and TGF-β.

12. Line 485: It would be interesting to examine markers of intestinal barrier function such as tight junction proteins and other factors that could impact intestinal permeability. Do authors have any plans on doing so?

**Reply**: Yes. We have plans to perform it. Schistosomiasis japonica can cause intestinal granulomas and inflammation and ulceration of intestinal mucosal tissue, seriously affecting the integrity and permeability of the intestinal barrier. Therefore, it is necessary to perform markers of intentional barrier function in the nest step of experiment.

**Response to Reviewer 2**

The manuscript by Chen et al., titled “Multi-omics analyses of Bacillus amyloliquefaciens treated mice infected with Schistosoma japonicum reveal dynamic changes in the intestinal microbiome and its associations with host metabolism,” explores the role of Bacillus amyloliquefaciens in mitigating pathological injuries in mice infected with Schistosoma japonicum by modulating the intestinal microbiome and metabolome. This manuscript builds on a 2023 study by the same group, employing a more comprehensive multi-omics approach and extending the scope of analysis from microbial diversity to more detailed functional and metabolic descriptions. The authors demonstrate the potential of Bacillus amyloliquefaciens in modulating host metabolism, providing a potential new avenue for alleviating schistosomiasis symptoms and lesions through the administration of the bacterium as a probiotic. The study shows that treating mice with B. amyloliquefaciens prior to their infection with S. japonicum leads to alterations in the microbiome, metagenome, and metabolome, which are used to explain how this bacterium alleviates some symptoms of the parasitic infection. I found the findings interesting and consider this a strong continuation of the previous manuscript.

**Reply**: Thank you for your patient review and detailed comments on our manuscript. We sincerely believe that your feedback will greatly be beneficial to the quality of our manuscript. Based on your detailed revision suggestions, we have made modifications to the corresponding words and sentences in the manuscript, rephrased some sentences which were complex and difficult to understand, and thoroughly checked grammar and expression. We have also provided detailed description of the methods section. At the same time, we have rearranged the figures to reduce complexity and make them more readable. I hope the revised manuscript meets your expectations. Our modifications and answers to your question are as follows.

**General suggestions:**

Improve the readability of the manuscript by making sentences clearer and enhancing the overall phrasing. Many errors could have been caught with a thorough language proofreading. I have included several suggestions below to improve the manuscript, but I must emphasize that this is not an exhaustive list. The authors should make further improvements beyond my suggestions, potentially hiring professional proofreading services.

**Reply**: Thank you for your valuable suggestion. We have thoroughly checked the grammar and expression of the manuscript. We have corrected incorrect grammar and unreasonable expressions to improve the readability of the article.

**Discussion section suggestions:**

I think that the Discussion section of the manuscript repeatedly presents the results and includes too many speculative propositions, making it difficult to follow. Additionally, it should cite figures when talking about the findings in this study. Overall, the section needs improvement to effectively communicate the contributions and implications of the study in a broader context.

**Reply**: Thank you so much for your valuable suggestion. We fully agreed with your viewpoint. Based on your suggestion, we have made revisions to the discussion. We have reduced the repetitive presentation of the results and removed unreasonable speculative statements to minimize misleading readers and increase the readability of the article. Meanwhile, we added citation to the figure when discussing the results. Please refer to the discussion section of our manuscript for detailed information.

**Conclusion section suggestions:**

The section repeatedly mentions the intervention of B. amyloliquefaciens, which could be streamlined for better readability. The conclusions are not clearly distinguished from the results and discussion, making it harder to discern the key takeaways. The conclusions could provide more insight into the implications and future directions of the research.

**Reply**: Thanks for your suggestions about our conclusion section. We fully agree with your evaluation of the conclusion section of our manuscript. Based on your suggestion, we have simplified the expression of the intervention of *B. amyloliquefaciens* and reduced its frequency of occurrence to improve readability. Meanwhile, we have made significant revisions to the conclusion section. We briefly summarized the results and provided an outlook on future directions based on the results, as well as a statement on the significance of the research. Please see line 587-599: “In conclusion, multi-omics sequencing technology was used in this study to jointly analyze the changes in gut microbiota and serum metabolites in mice infected with S. japonicum after intervention with B. amyloliquefaciens. We found that the metabolic pathways of gut microbiota may significantly affected the composition and alternation of serum metabolites in mice in response to intervention, thus potentially correlated with the alleviation of symptoms of schistosomiasis japonica (Fig 5). At the same time, some metabolites with effects of anti-inflammatory and antioxidant were identified through metabolomics analysis, which can be used as candidate substances for studying the alleviation of schistosomiasis japonica in the future, and will further clarify the mechanism by which B. amyloliquefaciens alleviates symptoms of schistosomiasis japonica. Our research demonstrated the potential probiotic mechanism of B. amyloliquefaciens, providing important references for precision drug design based on targeted gut microbiome, and offering new ideas for the prevention and adjuvant treatment of schistosomiasis japonica.”. I hope our revised manuscript can satisfy you.

**Line-specific suggestions:**

Line 19. "the intestine"

**Reply**: We have changed “intestine” to “the intestine”. Please refer to line 19: “the intestine”.

Line 20: "Probiotic therapy has been shown to be effective"

**Reply**: We have changed “Probiotic therapy shown to be effective” to “Probiotic therapy has been shown to be effective”. Please refer to line 20: “Probiotic therapy has been shown to be effective”.

Line 23: "unknown"

**Reply**: We have changed “unknow” to “the unknown”. Please refer to line 23: “the intestine”.

Line 26: "B. amyloliquefaciens was found to significantly"

**Reply**: We have changed “*B. amyloliquefaciens* were found to significantly” to “*B. amyloliquefaciens* was found to significantly”. Please refer to line 26: “*B. amyloliquefaciens* was found to significantly”.

Line 29: "glycerophospholipids"

**Reply**: We have changed “glycerophospholipid” to “glycerophospholipids”. Please refer to line 29: “glycerophospholipids”.

Lines 29-30: "and were shown to be significantly positively correlated"

**Reply**: We have changed “and shown to be significantly positively correlated” to “were shown to be significantly positively correlated”. Please refer to line 30: “were shown to be significantly positively correlated”.

Line 42: "as a potential strategy to alleviate"

**Reply**: We have changed “as potential strategy to alleviate” to “as a potential strategy to alleviate”. Please refer to line 42: “as a potential strategy to alleviate”.

Line 44: Abbreviate Bacillus amyloliquefaciens to B. amyloliquefaciens from here to the end of the text.

**Reply**: We have changed “*Bacillus* *amyloliquefaciens*” to “*B. amyloliquefaciens*” from line 44 to the end of the text.

Line 44: "schistosomiasis japonica through the mediation of the intestinal microbiome"

**Reply**: We have changed “schistosomiasis japonicum through the mediation of intestinal microbiome” to “schistosomiasis japonica through the mediation of the intestinal microbiome”. Please refer to line 44: “schistosomiasis japonica through the mediation of the intestinal microbiome”.

Line 46: "joint analysis of the gut microbiome and host serum"

**Reply**: We have changed “joint analysis of gut microbiome and host serum” to “joint analysis of the gut microbiome and host serum”. Please refer to line 45-46: “joint analysis of the gut microbiome and host serum”.

Line 47: Please clarify what does “intervention” mean here?

**Reply**: The “intervention” here means intragastric administration. We have changed “intervention” to “intragastric administration of *B. amyloliquefaciens*”. Please refer to line 47: “intragastric administration of *B. amyloliquefaciens*”.

Line 47: "intestinal microbiome and the secretion of metabolites derived from the intestinal microbiome"

**Reply**: We have changed “intestinal microbiome and the secretion of derived metabolites derived from intestinal microbiome” to “intestinal microbiome and the secretion of metabolites derived from the intestinal microbiome”. Please refer to line 48: “intestinal microbiome and the secretion of metabolites derived from the intestinal microbiome”.

Line 49: Rephrase for clarity: "improve lipid metabolism disorder and hormone secretion in mice"

**Reply**: We have changed “improve the disorder of lipid metabolism and hormone secretion of mice” to “improve lipid metabolism disorder and hormone secretion in mice”. Please refer to line 49: “improve lipid metabolism disorder and hormone secretion in mice”.

Line 50: “This study highlights the crosstalk between the intestinal …”

**Reply**: We have changed “This study highlighted the crosstalk between intestinal” to “This study highlights the crosstalk between the intestinal”. Please refer to line 50: “This study highlights the crosstalk between the intestinal”.

Line 60: Consider clarifying "Schistosoma japonicum is a type of schistosomiasis" to "Schistosoma japonicum is a parasitic worm that causes schistosomiasis."

**Reply**: We have changed “*Schistosoma* *japonicum* is a type of schistosomiasis” to “*Schistosoma* *japonicum* is a parasitic worm that causes schistosomiasis”. Please refer to line 59: “*Schistosoma* *japonicum* is a parasitic worm that causes schistosomiasis”.

Lines 65-66: Improve this sentence for clarity: “Pathological symptoms are the host response to the mechanism of autoimmune response.”

**Reply**: We have changed “Pathological symptoms are the host response to the mechanism of autoimmune response” to “Pathological symptoms are the host’s response to the autoimmune response”. Please refer to line 66: “Pathological symptoms are the host’s response to the autoimmune response”.

Line 74: I suggest rephrasing this sentence to read something like: "Gut microbiota is a diverse community of microorganisms that reside in the digestive tracts of humans and other animals."

**Reply**: We have changed “Intestinal microbiota is a complex community living in the human colon” to “Gut microbiota is a diverse community of microorganisms that reside in the gastrointestinal tract of humans and other animals”. Please refer to line 74-75: “Gut microbiota is a diverse community of microorganisms that reside in the gastrointestinal tract of humans and other animals”.

Line 82: “aggravates”

**Reply**: We have changed “aggravated” to “aggravates”. Please refer to line 83: “aggravates”.

Line 88: Improve this sentence for clarity: “Bacillus subtilis is the first microbe applied to schistosomiasis japonica.” What does this mean? During medical treatment in humans? In mice? Please clarify.

**Reply**: Thanks for pointing out this problem. Actually, *Bacillus* *subtilis* is the first microbe using in mice model. We have changed “*Bacillus* *subtilis* is the first microbe applied to schistosomiasis japonica” to “*Bacillus* *subtilis* is the first microbe applied to mouse model of schistosomiasis japonica”. Please refer to line 88-89: “*Bacillus* *subtilis* is the first microbe applied to mouse model of schistosomiasis japonica”.

Line 95: Why are the authors using “excellent” here? In other words, can the author inform which positive probiotic qualities.

**Reply**: We used inappropriate words here. We have changed “excellent” to “ideal”. Please refer to line 95: “ideal”.

Lines 104-107: Improve this sentence for clarity: “Through the analysis of intestinal microbiota and serum metabolites of host, the effectiveness of B. amyloliquefaciens intervention was evaluated, and the mechanism of schistosomiasis japonica relief was elaborated from the perspective of microbiome-host interaction.”

**Reply**: We have changed “Through the analysis of intestinal microbiota and serum metabolites of host, the effectiveness of B. amyloliquefaciens intervention was evaluated, and the mechanism of schistosomiasis japonica relief was elaborated from the perspective of microbiome-host interaction.” to “Through the analysis of intestinal microbiome and serum metabolites of host, we want to evaluate the effectiveness of *B. amyloliquefaciens* intervention, and elaborate the mechanism of schistosomiasis japonica relief from the perspective of microbiome-host interaction.”. Please refer to line 105-107: “Through the analysis of intestinal microbiome and serum metabolites of host, we want to evaluate the effectiveness of *B. amyloliquefaciens* intervention, and elaborate the mechanism of schistosomiasis japonica relief from the perspective of microbiome-host interaction.”.

Lines 111-120:

- Specify the bacterial strain used in the study, including a reference to the relevant genome or database.
- Provide the same information for the Schistosoma japonicum strain.
- Although previously reported, include a brief description of the culturing and growth conditions for both the bacterial and parasite strains.

**Reply**: Thanks for your valuable suggestions. We have provided the strain information of *B. amyloliquefaciens* and *S. japonicum*, and improved the description about the culturing and growth conditions for both the bacterial and parasite strains. Please refer to line 111 and line 114-117 for information and database about bacterial strain (“*B. amyloliquefaciens* strain 802”, “Detailed strain information of *B. amyloliquefaciens* strain 802 could be referred to National Center for Biotechnology Information (https://www.ncbi.nlm.nih.gov/) with accession of MT585518.1, and reference genome could be found at https://www.ncbi.nlm.nih.gov/datasets/genome/GCF_019396925.1/.”), line 118 for strain information about *S. japonicum* (“the Chinese mainland strain of *S. japonicum*”), line 112-113 for culturing and growth conditions for the bacterial (“Briefly, the bacterial strains were cultured in lysogeny broth medium and grew at 37 °C in the constant-temperature shaker with 200 rpm”), line 117-120 for culturing and growth conditions for *S. japonicum* (“Cercariae came from the Chinese mainland strain of *S. japonicum* which was maintained by serial passage through *O. hupensis* snails and the inbred Chinese Kun-ming mice. And snails were maintained in a simulated natural microenvironment at 23-25 °C, 12:12 h light:dark cycle and fed lettuce as needed for their breeding”).

Line 122-123: It appears that the authors are extracting total blood via the retro-orbital plexus route, as collecting whole blood from eyeballs is not feasible. If this was the procedure used, it typically requires anesthesia in mice. Please clarify why anesthesia was not mentioned or used in this case.

**Reply**: Thanks for pointing out this problem. We have used inappropriate and incorrect expressions in the description of the blood collection here, which caused confusion for you. We have revised it into “Mice from groups of SJ and SJBA were performed to collect serum samples on day 45. After anesthesia with isoflurane, blood samples were drawn from orbital veins and sacrificed mice subsequently by cervical dislocation.” Please refer to line 137-139.

Line 136: There is no mention of any barcodes before here. Describe the library design, including where these barcodes are located.

**Reply**: Thanks for pointing out it. We have improved description about this. Please refer to line 151-161: “Sample-specific 7 bp barcodes were incorporated into the primers for multiplex sequencing. The PCR components contained 5 μL of Q5 reaction buffer (5×), 5 μL of Q5 High-Fidelity GC buffer (5×), 0.25 μL of Q5 High-Fidelity DNA Polymerase (5U/μL), 2 μL (2.5 mM) of dNTPs, 1 μL (10 μM) of each Forward and Reverse primer, 2 μL of DNA Template, and 8.75 μL of ddH2O. Thermal cycling consisted of initial denaturation at 98 °C for 2 min, followed by 25 cycles consisting of denaturation at 98 °C for 15 s, annealing at 55 °C for 30 s, and extension at 72 °C for 30 s, with a final extension of 5 min at 72 °C. PCR amplicons were purified with Agencourt AMPure Beads (Beckman Coulter, Indianapolis, IN) and quantified using the PicoGreen dsDNA Assay Kit (Invitrogen, Carlsbad, CA, USA). After the individual quantification step, amplicons were pooled in equal amounts, and paired-end 2×300 bp sequencing was performed using the Illumina NovaSeq platform with the NovaSeq-PE250 sequencing strategy at Shanghai Personal Biotechnology Co., Ltd. (Shanghai, China).”.

Line 159: May be change to “Metabolomic profiling”?

**Reply**: We have changed “Metabolomic sequencing of serum metabolites” to “Metabolomic profiling of serum metabolites”. Please refer to line 194: “Metabolomic profiling of serum metabolites”.

Line 185-186. Rephrase to make it more clear: “Heatmap presented the number of genus belonged to different occurrence patterns …”

**Reply**: We have changed it into “And heatmap was used to present the number of genus-level bacteria in three occurrence patterns which was performed by R package “pheatmap””. Please refer to line 219-220.

Line 205. “and annotated to HMDB and KEGG.”

**Reply**: We have changed “and annotated to HMDB and KEGG” to “to be annotated using HMDB and KEGG”. Please refer to line 239: “to be annotated using HMDB and KEGG”.

Line 219: I believe it is called “Random Forest”. Change it in the rest of the manuscript if you agree.

**Reply**: We have changed it into “Random Forest” from here to the rest of the manuscript.

Line 227: “orange lines”. Additionally: The end of the sentence seems to be missing. To show what?

**Reply**: Thanks for your suggestion. We believed that “orange lines” was absolutely correct. However, based on the feedback from other reviewers, we have made revisions to the figures and correspondingly modified the corresponding the content of methods section. So, we deleted this content.

Lines 236-238: For clarity, rephrase the whole sentence starting with: “According to results …”

**Reply**: We have changed it into “According to results of the previous research, the intervention of *B. amyloliquefaciens* significantly alleviated the pathological symptoms of mice infected with *S. japonicum*”. Please refer to line 274-275.

Line 238: “to detect the impact”

**Reply**: We have changed it into “to detect the impact”. Please refer to line 275.

Line 238: S1 Fig is not a result from this study, but from their previous 2023 manuscript. For me this does not make sense to include it here as this manuscript can be cited.

**Reply**: We completely agreed with your viewpoint. We have deleted **S1 Fig** and used it as citation.

Line 242-244: “to cluster 3” … “to cluster 2”. “Additionally, the microbial community of cluster 1 was distinct from that of cluster 3”

**Reply**: We have changed these you mentioned. Please refer to line 279-282: “Based on the importance of bacterial genera to accuracy of models, *Escherichia Shigella* had the highest contribution to cluster 3, while *Muribaculaceae* contributed more to cluster 2. Additionally, the microbial community of cluster 1 was distinct from that of cluster 3”.

Line 249: Avoid using tremendously, instead use something like “was significantly altered”.

**Reply**: We have changed it into “altered significantly”. Please refer to line 287.

Line 256-259: For clarity, rephrase sentence starting with “Intersection of genera …”

**Reply**: We have rephrased it into “Results of intersection analysis of bacterial genera belong to “Core” occurrence pattern showed that among the 18 bacterial genera present simultaneously in four groups, *Escherichia Shigella*, *Helicobacter* and *Lactobacillus* increased on day 45 in SJ group while *Parabacteroides, Erysipelatoclostridium* and *Corynebacterium* increased on day 45 in SJBA group”. Please refer to line 294-297.

Line 275: “in mice”

**Reply**: We have modified it into “in mice”. Please refer to line 306-307.

Line 302: “..., and so on (Fig 2E)”. I would suggest avoiding being vague here. You might want to highlight here which ones are important to this study and potentially why.

**Reply**: Thanks for your suggestion. We deleted the “so on” in line 335.

L321. “with B. amyloliquefaciens”

**Reply**: We have changed “of *B. amyloliquefaciens*” into “with *B. amyloliquefaciens*”. Please see line 354.

L331. "accounts for high proportions". Write it more clearly.

**Reply**: We have changed it into “were also top five in terms of proportion”. Please refer to line 365.

L332. “From the 494 HMDB metabolites, 226 were annotated in the KEGG database and were used”

**Reply**: We have changed it into “Furthermore, 226 out of the 494 differential HMDB metabolites were annotated using the KEGG database”. Please refer to line 365-366.

L372. Improve the sentence “Metabolism of amino acids and lipids was found to be more significant…” by completing the metric that is being referenced. What is more significant?

**Reply**: We have improved it into “Suggested by the Figure 3D, metabolism of amino acids and lipids was found to be more important in the infected mice after treating with *B. amyloliquefaciens* for their higher proportions of metabolic pathways with significant changes.”. Please refer to line 393-395.

L375. “higher”.

**Reply**: We have improved it into “higher”. Please refer to line 397.

L390. “It is worth noting”

**Reply**: We have improved it into “It is worth noting”. Please refer to line 412.

L401. Add punctuation to the sentence.

**Reply**: Thanks for your suggestion. We admitted that this sentence was indeed very long and difficult to understand, and punctuation was necessary to be added. However, we have deleted this sentence for other reviewers’ comments on figures and results.

Line 415. “Boxplot presents”.

**Reply**: We have changed it into “Boxplot presents”. Please refer to line 430.

L423. belonging/that belong

**Reply**: Thanks for your suggestion. We have deleted this sentence during the revision.

Line 431: "enables it to endure the low pH of gastric juice" - should be rephrased to "enables it to withstand the low pH of gastric juice".

**Reply**: We have changed “enables it to endure the low pH of gastric juice” into “enables it to withstand the low pH of gastric juice”. Please refer to line 446-447.

Line 435: "was gavage to mice infected with" - should be "was administered via gavage to mice infected with".

**Reply**: We have changed “was gavage to mice infected with” into “was administered via gavage to mice infected with”. Please refer to line 450.

Line 438: "the niche of the intestinal microbiota can remain stable and undisturbed" - should be rephrased to "the niche of the intestinal microbiota remains stable and undisturbed".

**Reply**: We have changed “the niche of the intestinal microbiota can remain stable and undisturbed” into “the niche of the intestinal microbiota remains stable and undisturbed”. Please refer to line 453.

Line 442: "has always maintained state of dynamic balance" - should be rephrased to "has always maintained a state of dynamic balance".

**Reply**: We have changed “has always maintained state of dynamic balance” into “has always maintained a state of dynamic balance”. Please refer to line 457.

Line 447: "one of the effectiveness of B. amyloliquefaciens intervention" - should be "one of the effects of B. amyloliquefaciens intervention".

**Reply**: Thanks for your suggestion. We have deleted this sentence.

Line 450: "which can only be the death and disappearance as final result" - should be rephrased to "resulting in the death and disappearance of other core bacterial genera".

**Reply**: We have changed “which can only be the death and disappearance as final result” into “resulting in the death and disappearance of other core bacterial genera”. Please refer to line 462-463.

Line 451: "the influence of Escherichia Shigella" - should be "the impact of Escherichia Shigella".

**Reply**: We have changed “the influence of Escherichia Shigella” into “the impact of *Escherichia Shigella*”. Please refer to line 463.

Line 454: "reached another relatively stable equilibrium state" - should be "reached a different but relatively stable equilibrium state".

**Reply**: We have changed “reached another relatively stable equilibrium state” into “reached a different but relatively stable equilibrium state”. Please refer to line 466.

Line 456: "be used as carbon source" - should be "be used as a carbon source".

**Reply**: We have changed “be used as carbon source” into “as a carbon source”. Please refer to line 468.

Line 458: "to kill E. coli" - should be "to kill Escherichia coli".

**Reply**: We have changed “to kill E. coli” into “to kill *Escherichia* *coli*”. Please refer to line 470.

Line 459: "But the specific mechanism still" - should be "However, the specific mechanism still".

**Reply**: We have changed “But the specific mechanism still” into “However, the specific mechanism still”. Please refer to line 471.

Line 440: I would specify where this statement comes from "... composition and abundance of intestinal microbiota fluctuated during the experiment X, the core microbiota containing key species remained stable (Fig X)."

**Reply**: Thanks for your suggestion. Actually, it’s not our results from this study. This is the conclusion from others’ research. To avoid misleading readers, we have added a reference to this sentence. Please refer to line 456.

Line 443. I would rephrase "the immense change of intestinal microbiota was observed to be inhibited after intervention of B. amyloliquefaciens, so that the ecology of intestinal microbiota remained stable in another state." Instead, I would change it to something similar to "the observed change in intestinal microbiota was inhibited following the intervention of B. amyloliquefaciens, as well as stabilizing the ecology of the intestinal microbiota in another state."

**Reply**: Thanks for your suggestion. In order to avoid repeatedly stating the results, we have deleted this sentence and changed it into “Through the establishment of DMM model and the definition of different occurrence patterns, intestinal dysbacteriosis and the growth of *Escherichia Shigella* were found to be inhibited after intervention of *B. amyloliquefaciens*”. Please refer to line 458-460.

Line 435: Change this sentence as it is misleading: “B. amyloliquefaciens was gavage to mice infected with S. japonicum.”. Describe the experiment detailed in Methods, as B. amyloliquefaciens was gavage to mice one week before the infection with S. japonicum..

**Reply**: We have changed it into “*B. amyloliquefaciens* was administered via gavage to mice infected with *S. japonicum*.” (line 450). And we have added the description about the detail in methods. Please refer to line 130-133: “Intragastric administration via oral gavage of PBS and *B. amyloliquefaciens* (1×10^9^ CFU/mL) was conducted every three days, starting from one week before the infection till the day of mice sacrifice. The dose of each intragastric administration was 0.3 mL. All mice were sacrificed on day 45.”.

Line 459: This sentence is speculative. It should not be a fact stated but rather a plausible explanation: Something like “Therefore, B. amyloliquefaciens could be alleviating the dysbiosis of microbiota and remodeled the homeostasis of gut by regulating the competition of microbiota in the niche and its nutrient composition.”

**Reply**: We have changed it into “Therefore, *B. amyloliquefaciens* could be alleviating the dysbiosis of microbiota and remodeling the homeostasis of gut by regulating the competition of microbiota in the niche and its nutrient composition.”. Please refer to line 468-470.

Line 476-479: Clarify in which model it confers protection.

**Reply**: Thanks for your suggestion. Based on your suggestion, we have added the models into this sentence which others’ researches used. Please refer to line 488-490: “such as in six-week-old male A/J mouse models treated with different chow diets laced with *P.* *distasonis* or Dextran Sulfate Sodium Salt (DSS)-induced BALB/c mice with oral treatment of *P.* *distasonis*”.

Line 480: Genus in italics.

**Reply**: We have changed it into “*Alistipes*”. Please refer to line 491.

Line 527: “play”

**Reply**: Thanks for your suggestion. However, we have deleted this sentence.

Lines 577-578: Follow Line 435 suggestion.

**Reply**: We have changed it into “intragastric administration of *B. amyloliquefaciens*”. Please refer to line 567.

Lines 588-589: “In this study, we found that various metabolic pathways (steroid hormone biosynthesis, arachidonic acid metabolism, glycerophospholipid metabolism) are carried out in the liver.” Where have the authors demonstrated this?

**Reply**: Thanks for pointing out this problem. This is our statement error. What we wanted to express was that some metabolic pathways could be carried out in the liver according to others researches. We have changed it into “The various metabolic pathways identified in this study (steroid hormone biosynthesis, arachidonic acid metabolism, glycerophospholipid metabolism) could also be carried out in the liver.” Please refer to line 573-575.

**Figure suggestions:**

Fig 3. I suggest writing a more meaningful figure title, for example, instead of just mentioning cluster 1 and cluster 3, referencing the treatment that gives rise to these clusters. Panel (C) has too many details to be clearly seen for its size. I would suggest making a bigger plot and move it as a supplementary figure or reconfigure Fig 3, reducing panel (D) and maybe trimming the plot on (E). (E) Revise the phrasing for clarity.

**Reply**: Thanks for your suggestion on figures. We have modified the title of Figure 3 into “Treatment of *B. amyloliquefaciens* significantly altered serum metabolites in the mice infected with *S. japonicum* on day 45.” (line 380). And we have move panel (C) into supplemental materials. Then we trimmed panel (E) (it was panel (D) now after revision) to make it look more concise. Finally, we have rearranged Figure 3 to make it look more concise and readable overall. Please refer to figure below and Figure 3 in the manuscript.

Figure 4. Types are too small. I suggest breaking it up into two or more figures. Panel A, explain where the data comes from.

**Reply**: Thanks for your suggestion. In order to reduce the complexity of Figure 4, we have rearranged it without changing the results basically. We have summarized panel (B) and presented it in more concise panel. Original panel (B) was moved into supplemental materials. And we moved histogram from panel (C) into supplemental materials. As for panel (D), we have reduced the content and visualized the correlation between microbes, genes and metabolites according to different groups to improve the readability of the charts. Finally, we have separated the small panel in gray dashed box of the original panel (D) and renamed them as panel (E). And it has been streamlined, highlighting the changes in metabolic pathways that were important in cluster 1 group. Please refer to figure below and Figure 4 in the manuscript for detailed information. Data from panel (A) was obtained from metagenomics analysis. Please refer to line 390-393 for detailed description: “Suggested by the **Figure 3D**, metabolism of amino acids and lipids was found to be more important in the infected mice after treating with *B. amyloliquefaciens* for their higher proportions of metabolic pathways with significant changes. So, differential genes related to these two metabolic pathways were screened by metagenomic analysis. The abundance of 24 differential genes were presented”.

Figure 2. I suggest eliminating the dots color code and change it to cluster number next to the species (blue and turquoise are too similar, and red gets confused with abundance heatmap dots. Otherwise, picking different colors. Additionally, moving Kegg footnote reference to fit panel (E).

**Reply**: Thanks for your suggestion. We have changed the colors of the dots into brown, purple and green and added black silhouette to highlight them. And we also rearranged Figure 2 and moved KEGG footnote reference to fit panel (E). Please refer to figure below and Figure 2 in the manuscript.

Figure 5. This figure has many elements that cannot be read clearly, such as barely visible numbers on the bar plots. It is complex and not self-explanatory, and the figure legend does not provide sufficient guidance to the reader. My suggestion is to make it clearer in every sense possible.

**Reply**: Thanks for your suggestion on this figure. In order to reduce the complexity of the figure, we have reduced the elements. We have reduced the types of metabolites mentioned in Figure 5 and only included the metabolites mentioned in Figure 4. At the same time, we increased the size of numbers on the bar plots. Furthermore, we have provided detailed explanations of all elements in Figure 5 in the legend to improve the readability of the figure (refer to line599-610: “**Fig 5. Potential mechanisms by which dynamic changes of intestinal microbiome affect serum metabolites in mice infected with *S. japonicum* after intervention of *B. amyloliquefaciens*.** Red in pie plot and histogram plot represented cluster 3 while blue represented cluster 1. The patterns in the background were intestine, which meant that metabolic pathways derived from gut microbiota could influence serum metabolites in blood vessels. Red up-arrows represented significant enrichment of microbes or genes or metabolites in cluster 1 compared to cluster 3 while blue down-arrows represented significant depletion. Full-line arrows showed that serum metabolites sourced from microbiota could be generated by gut microbiota-derived metabolic pathways mediated by functional genes. Dashed-line arrows meant that serum metabolites from different source had strong correlation with gut microbiota, but can’t find any microbiota-derived relevant differential functional genes”). Please refer to figure below and Figure 5 in the manuscript.

**Response to Reviewer 3**

**Parts of Methods**

1. Line 117: Why were the mice infected with 27±3 cercariae, whereas the usual number is 12 or 20?

**Reply**: Thanks for your question. When designing the experiment, we referred to the number of cercariae in this article (doi: 10.3389/fimmu.2020.569727), so we chose 27±3 as the infectious dose.

2. Line 118: The administration of B. amyloliquefaciens started one week before SJ infection. Normally, the treatment/intervention/eradication should be applied to the models after infection. Why was this timing chosen?

**Reply**: Thanks for your question. There was one review indicating the potential application of probiotics in schistosomiasis endemic areas (doi: 10.1186/s13071-024-06245-1). This also gave us some inspiration. We speculated whether pre-intervention of *B. amyloliquefaciens* to susceptible populations or healthy populations living in schistosomiasis endemic areas can reduce schistosomiasis infection or alleviate the severity of post infection symptoms. Therefore, we chose to pre-intervention of *B. amyloliquefaciens* before infection.

3. Line 123: Why were serum samples collected from the eyeballs instead of the tail vein? The blood-retinal barrier may filter certain metabolites, and this method does not meet the animal ethics requirement.

**Reply**: Thank you for pointing out the error in our statement. In fact, what we really wanted to express was taking blood from orbital veins. We were well aware that taking blood from the eyeball didn’t meet the animal ethics requirement. We forgot to make modifications to this section while writing the methods. Therefore, based on your suggestion, we have improved the content of the methods section and provided a detailed explanation of our blood collection method. Please refer to line 134-136: “Mice from groups of SJ and SJBA were performed to collect serum samples on day 45. After anesthesia with isoflurane, blood samples were drawn from orbital veins and sacrificed mice subsequently by cervical dislocation.”.

4. Line 126 and 140: According to lines 112-116, there should be 7 mice in each group. Were stool samples collected from only 3 out of the 7 mice? Additionally, in line 160, it is mentioned that serum samples were collected from 6 out of 7 mice. What were the criteria for selecting these samples? Please clarify.

**Reply**: Thanks for raising this question. In order to meet the requirements of biological reproducibility and statistical analysis of data, we randomly selected 3 samples from each group for sequencing. Meanwhile, due to the significant differences in the composition of serum metabolites among each mouse, in order to ensure data consistency and reduce the impact of individual differences, we randomly selected 6 samples from each group for metabolomics sequencing. When selecting these samples, in order to reduce subjectivity, we randomly selected the samples. And we have added “randomly selected” into methods.

5. Line 137: Reference #131 summarizes pipelines for the analysis of amplicon and metagenomic data. The author should list the specific tools/methods used in this work.

**Reply**: Thanks for your suggestion. Based on your suggestion, we have listed specific tools used for the analysis of amplicon in the methods. Please refer to line 159-165: “The raw reads after sequencing were decomposed into appropriate samples based on the barcodes and then imported into the Quantitative Insights into Microbial Ecology (QIIME2) [33]. Plugin DADA2 denoise-paired was used to dereplicate the raw data and create a feature table and feature representative sequences [34]. Plugin feature-classifier and classify-consensus-blast were used to perform taxonomic classification based on Silva 138 reference sequence (MD5: a914837bc3f8964b156a9653e2420d22) and taxonomy files (MD5: e2c40ae4c60cbf75e24312bb24652f2c) [35]. Plugin taxa were used to remove non-bacterial sequences and mitochondrial chloroplast contamination.”.

6. Line 150: Bowtie2 was used for alignment and which tools/script were used for filtration? Line 152: The compositions of human and mouse gut microbiotas are distinct. Why was MGBC (doi: 10.1016/j.chom.2021.12.003) not used?

**Reply**: Thanks for your suggestions. Bowtie2 was used to align the raw reads into host reference genome to remove reads from host. So, the host-free reads were obtained and host reads were filtered after using Bowtie2. We have modified the description about it in line 178-181: “Then, in order to filter out the sequence from mice, Bowtie2 was used to align the processed raw reads to the reference genome of mice (mm39) (https://www.ncbi.nlm.nih.gov/datasets/genome/GCF_000001635.27/) and host-free reads could be obtained subsequently by specifying --un-conc” parameter”. And using “UHGG” was our mistakes in writing, we forgot to change it to the database we used in this analysis. We have revised our expression in line 182-183: “based on the Mouse Gastrointestinal Bacteria Catalogue (MGBC) database”.

7. Line 155: Salmon can quantify reads, but how to estimate the coverage?

**Reply**: This is a good question. We have constructed gene catalog by script “Salmon index” and then mapped raw reads into gene catalog to estimate the read coverage. We have improved this description in detail, and please refer to line 186-188: “The read coverage for each gene in different samples was estimated and normalized through Salmon by mapping raw reads from each sample to sequences in gene catalog constructed by script “Salmon index””.

8. Fig. 1B: There should be around 15 time-points as the samples were collected every 3 days. Why was these 5 sampling time points chosen?

**Reply**: Thanks for pointing out this problem. In fact, we did collect fecal samples every three days, but due to funding issues, we chose suitable 12-day interval for sequencing. “Five sampling time points” were day 0, day 12, day 24, day 36 and day 45 after infection of *S. japonicum*, respectively. We wanted to observe the dynamic changes of gut microbiota over time, and 12 days as an interval is neither long nor short, which was very suitable. The reason why day 45 infection of S. japonicum were selected to be one sampling time point was that because the model mice were on the brink of death due to worsening symptoms of schistosomiasis on day 45, so we had to collect samples and sacrifice mice. That was the reason why we chose five sampling time points. We have improved the description of it, please refer to line 143-144: “Three stool samples from each of the four groups at five sampling time points (day 0, day 12, day 24, day 36, day 45 after infection of S. japonicum) were selected for 16S rRNA gene sequencing (Fig 1A).”.

9. Fig. 1 and 2: Were the three DMM clusters in Fig. 2 (metagenomic sequencing data) carried over from Fig. 1 (16S)? If so, the dimensionality reduction methods (NMDS or PCoA) should be consistently applied to 16S (Fig. 1A) and the further metagenomic seq data (Fig. 2A).

**Reply**: Thanks for pointing out this problem. We confirmed that the three DMM clusters in Fig. 2 (metagenomic sequencing data) carried over from Fig. 1 (16S). And we have modified the analysis methods to make sure that the dimensionality reduction methods were consistently applied to 16S (Fig. 1B) and the metagenomic seq data (Fig. 2A). Please refer to the figures below (left is Fig 1B and right is Fig 2A) and in the manuscript.

10. How was the normalization of sequencing data performed?

**Reply**: Thanks for pointing out this question. These was our negligence in not specifying how to do it in the method. We used several methods to perform the normalization of sequencing data. Bracken was used to normalize the abundance of bacterial species. Salmon was used to estimate the read coverage of gene and convert reads count to TPM to normalize the abundance of gene. And abundance of species or genes was flatted before performing beta analysis by using “vegan” package. We have added these descriptions into manuscript. Please refer to line 183 (“and relative abundance was normalized by using Bracken based on the MGBC database”), line 187 (“normalized through Salmon”), line 222 (“Abundance flatting following by Nonmetric Multidimensional Scaling (NMDS) analysis”).

**Parts of Results**

1. Line 249: How was this 'suggestion' derived? The description for SJBA group should be added.

**Reply**: Yes, we have added the description about SJBA in line 283: “But the community types of SJ group switched to cluster 3 on day 45 while SJBA group still remained cluster 1.”.

2. Fig.1E: Is it including all genera list in figure 1C-’Disapperrance’ or as the description in line 259, only including those significantly disappear in any groups that were presented from day zero to 36, but decreased or even got eliminated on day 45, which might be the first two rows? Please clarify in the text.

**Reply**: Thanks for pointing out this. Actually, it was all bacterial genera listing in Fig 1C-“Disapperance” pattern. We have improved it in methods in line 218: “Intersection plots represented intersection of all bacterial genera belong to “Core” or “Disappearance” occurrence patterns”.

3. The color codes for clusters 1 and 2 are hard to distinguish, especially in Fig. 2C.

**Reply**: We agreed with your comments. So, we have changed colors code. Please refer to figures below and in the manuscript.

4. Fig. 2B and 2C: '33 representative species were selected by overlapping species set' and shown in Fig. 2C, including 'Unknown species.' What are the six species solely in the 'differential species' category?

**Reply**: The taxonomic annotation at the genus level of “Unknown species” was *Methanomassiliicoccus*, but the specific annotation information at the species level was “*Methanomassiliicoccus* unknown species” which meant that we knew it can annotate at the species level of this bacterial genus, but the specific naming was still pending identification. So, we used “Unknown species’’. And the six species meant that these species could not annotate to the species level, which their annotation information at the species level was blank, so we thought that the six species belonged to that part. We didn’t know their specific information at the species level, but the highest classification level they belonged to included Phocaeicola (Genus), Bacteroidota (Phylum), Actinobacteriota (Phylum), Bacteroidaceae (Family), Muribaculaceae (Family), Firmicutes (Phylum).

5. Line 295: ‘Escherichia coli and Bacteroides stercorirosoris were found to be significantly increased in cluster 3, while Alistipes dispar and Parabacteroides distasonis were found to be significantly increased in cluster 1, which was similar to the analysis results of 16S rRNA gene sequencing (Fig 2C).’ The 16S analysis compared data within groups over time, whereas the metagenomic data compared different groups at a single time point. The importance of the similarity between them should be stated clearly.

**Reply**: What you were considered was right. We have changed our expression into “which was similar to the analysis results on day 45 of 16S rRNA gene sequencing” in line 327.

6. Line 299: ‘After mapping differential genes to the KEGG metabolic pathways, 20 metabolic pathways were found to be enriched after treatment with B. amyloliquefaciens’. Does this involve all 246 depleted and 1131 increased differentially abundant genes? If so, what is the biological implications of analyzing the depleted and increased genes together?

**Reply**: Yes, the 20 metabolic pathways involved all 246 depleted and 1131 increase genes. Our purpose in doing this was to demonstrate which metabolic pathways did these significantly enriched and depleted differentially expressed genes come from. And among these metabolic pathways, which ones have the most significant changes (enrich or deplete) in genes. Therefore, we chose this analysis method. We analyzed these genes together, focusing only on whether they significantly change, rather than whether they significantly enriched or depleted.

7. Line 326: ‘the NMDS result of the gut metagenome’ It is inaccurate and informal. Specify which results were consistent.

**Reply**: We have changed it into “which was consistent with the result of NMDS analysis in the gut metagenome that cluster 1 and cluster 3 was significantly distinct” in line 356-357.

8. Line 332: ‘226 metabolites annotated to the KEGG database based on the 494 differential metabolites’. should be ‘226 out of the 494 differential metabolites were annotated using the KEGG…’? And, clarify why 1023 differential metabolites were not annotated directly using KEGG, but had HMDB included as a filter step.

**Reply**: We have changed it into “226 out of the 494 differential metabolites were annotated using the KEGG” in line 363. The reason why we had HMDB included as a filter step was that some metabolites can be annotated by KEGG, but not by HMDB. And some metabolites can be annotated by HMDB, but not by KEGG. If we use these two databases as screening criteria simultaneously, the metabolites obtained will be more representative and the source and information will be clearer.

9. Fig. 3C: The figure legend mentions 'Pearson' analysis, while the text refers to 'Spearman'. Clarify which analysis was actually used. Additionally, the ‘33 differential species’ were abundant in only one of the clusters (1, 2, or 3). Either remove those from cluster 2 in analysis for Fig. 3C or clarify whether these metabolites were correlated with clusters 1 or/and 3 in the text.

**Reply**: Thanks for pointing out this problem. Actually, it was Spearman analysis. But we thought what you concerned was correct, and we deleted panel (C) in the Figure 3 to avoid misunderstanding readers. And we ensured what we do would not change and influence the results.

10. Line 376: ‘In order to clarify the cooperativity of the abundance changes of these markers between the two groups, Procrustes analysis was performed.’ This is the first time that ‘markers’ appears. Please clarify what ‘these markers’ include, what ‘two groups’ refer to, and what ‘Procrustes analysis’ was performed on. Also in line 378, specify which 'markers in bacteria, functional genes, and metabolites' were included.

**Reply**: What you mentioned was absolutely reasonable. Baased on your suggestions, we have added the definition of markers in the methods (line 247-249: “Markers were defined as differential intestinal microbes (33), differential functional genes from lipids metabolism and amino acid metabolism (24), and differential metabolites source from host or microbiota or both (71).”). “two groups”, “Procrustes analysis” and “markers in bacteria, functional genes, and metabolites” were also did the detailed descriptions. Please refer to line 396-399: “Procrustes analysis between abundance of markers (Refer to the Methods and Materials for definition) of microbes, functional genes and metabolites between cluster 1 and cluster 3 was performed in order to clarify the cooperativity of them. The results showed that the abundance changes of any two groups of markers in cluster 1 and cluster 3 were highly correlated”.

11. Line 380: ‘synergy’ refers to interactions among different components that result in effects greater than what would be expected from their individual actions. While Procrustes analysis can elucidate the correlations between different components within a single sample, it is insufficient to prove the synergy among ‘these makers’.

**Reply**: Thanks for explaining the meaning of the word. We agreed with your opinion and have deleted this word.

12. Line 381: ‘the representative species, genes, and metabolites before and after treatment with B. amyloliquefaciens’. Should ‘before and after treatment’ be ‘with and without treatment’? If so, what is the difference between ‘markers’ and the ‘representative species, genes, and metabolites’? Specifically, what species, genes and metabolites were applied to Random Forests models to screen for the ‘the representative species, genes, and metabolites’?

**Reply**: We have changed it into “with and without treatment with *B. amyloliquefaciens* in infected mice” in line 400. Representative microbes, genes, and metabolites were screened from markers. And markers were included differential intestinal microbes (33), differential functional genes from lipids metabolism and amino acid metabolism (24), and differential metabolites source from host or microbiota or both (71), which was applied to Random Forests models. We have improved the descriptions about these in the manuscript. Please refer to line 400 (“Then, Random Forest was used to identify the representative microbes, genes, and metabolites from markers with and without treatment with *B. amyloliquefaciens* in infected mice.”) and line 247-249 (“Markers were defined as differential intestinal microbes (33), differential functional genes from lipids metabolism and amino acid metabolism (24), and differential metabolites source from host or microbiota or both (71).”).

13. Fig. 4C: The x-axis title should be ‘% increase in mean squared error’.

**Reply**: Although we moved the part of Fig 4C into supplemental materials, we have changed the x-axis title into “% increase in mean squared error”.

14. Line 394: ‘lipid metabolism (like glycerophospholipid and arachidonic acid) had more positive correlation with species and genes.’ The interpretation is inaccurate as the Sankey plot showed the correlations between species and genes, and between genes and metabolites. Besides, it should be revised to ‘more metabolites involved in lipid metabolism correlated with…’.

**Reply**: We have changed “lipid metabolism (like glycerophospholipid and arachidonic acid) had more positive correlation with species and genes” into “”

15. Line 396: ‘metabolites from arachidonic acid metabolism were all enriched significantly in cluster 1’ How were this indicated by the spearman correlation analysis?

**Reply**: Thanks for pointing out this problem. Actually, we have deleted this sentence and modified the Figure 4D. In the previous writing, we marked the metabolites that significantly increased in cluster 1 with corresponding colors in Figure 4D. Meanwhile, you could also find evidence in the S8 Fig. However, based on the content of your next comment, we have made modifications to Figure 4D and accordingly made changes in the Results section. Therefore, we have removed this sentence

16. Line 397: The metabolic pathways were involved by enzymes, not ‘genes’. What kind of ‘sequential’ relationship do the gene-coded enzymes and metabolites show? Did the enzymes coded by tyrA2 and aroF, fabl and melA,ilvC and leuA catalyze the synthesis or degradation of tyrosol and cinnavalininate, 1-acyl-sn-glycero-3-phosphocholine and phosphatidylcholine, isopropylmaleic acid, 3-isopropylmalic acid? Besides, these genes and metabolites involving in same pathways in mice, irrespective of B. amyloliquefaciens intervention? What are the biological implications as the results were not interpreted for cluster 1 and cluster 3, respectively?

**Reply**: Thanks for pointing out this crucial problem. We agreed your opinion completely. Based on your evaluation, we understood that our analysis results in this section were not clear enough. Therefore, we made modifications based on this. Based on the results of correlation analysis between microbes to genes and genes to metabolites, we screened out the three (microbes, genes or metabolites) significantly enriched simultaneously in cluster1 or cluster3, and presented positive correlation in cluster1 or cluster3. Meanwhile, based on the results, we identified genes and metabolites belonging to the same reaction pathway and demonstrated their abundance changes between cluster 1 and cluster 3. And we have made corresponding changes in the figure, method and result in the manuscript.

**Parts of Conclusions**

1. Line 441: ‘Although the composition and abundance of intestinal microbiota have been fluctuating all the time due to external interventions such as diet and probiotics, the core microbiota containing key species remained stable.’ add citation.

**Reply**: We have added citation for this sentence. Please refer to line 454.

2. Line 453: ‘enhancing the colonization of other intestinal bacteria’. The term ‘other intestinal bacteria’ seems to refer to the ‘46 genera observed to significantly disappear in SJ group’ in Fig. 1D. To support the proposed ‘explanation’ in line 455, most of these disappeared genera should not be other pathogens/opportunistic pathogens. This information also provides evidence for the statement regarding the 'gradually recovered' 'beneficial bacteria' in line 474.

**Reply**: Thanks for this question. What you said was right. To observe the abundance of these disappearing genera mentioned in the SJ group in the SJBA group on day 45, we visualized their abundance (figure below). Result shown that the abundance of some beneficial genera in the intestine were relatively high, like *Roseburia, Blautia, Lachnoclostridium*, which could support our words.

3. Line 456: Add the full name of EPS. ‘EPS which can be used as carbon source by the intestinal microbiota, thereby crowding out pathogenic bacteria to inhibit their colonization and growth’ is not supported by ref #27. Correct the citation.

**Reply**: We have added the full name of EPS and added a new citation to support our opinion. Please refer to line 468.

4. Line 467: The high expression of intestinal pro-inflammatory cytokines induced by E. coli and those involved in the formation of intestinal granulomas are not the same. It is also unclear whether the changes in the intestinal environment caused by E. coli contribute to granuloma formation, or if the altered environment during the granuloma formation process due to S. japonicum infection provides an advantage for E. coli colonization. The causal relationship here is not well-defined, so the term "well proves" is too strong. The similar issue can be found through the discussion. Another example in line 484, the text presents a suggestion or hypothesis, as the improvement of the intestinal microenvironment or the regulation and repair of the intestinal barrier have not been investigated or validated.

**Reply**: What you concerned was right. So we have changed our expressions into “The inhibition of *E. coli*, which indicated the competitive exclusion effect of the microbiota interaction, may play important role in reducing the symptoms of mice infected with *S. japonicum.*” in line 481-483, “which could become one of the influencing factors of further development of intestinal granuloma caused by *S. japonicum*” in line 480-481 and “In a word, the structure and composition of intestinal microbiota in *S. japonicum* infected mice was improved after the intervention of *B. amyloliquefaciens*, which may play key role in slowing down the pathological symptoms and enhancing vital signs of infected mice.” in line 499-501 to avoid giving overly affirmative conclusions.

5. Line 484: What is meant by ‘granuloma infection’?

**Reply**: What we wanted to express truly was that infection of *S. japonicum* could induce intestinal granuloma. Then this symptom could cause intestinal inflammation and damage the integrity of the intestinal mucosa, leading to changes in the intestinal microenvironment. But we have deleted these words finally.

6. Line 487: Which ‘opportunistic pathogenic bacteria’ were been inhibited by intervention of B. amyloliquefaciens? The claim ‘intervention of B. amyloliquefaciens inhibited adverse intestinal microbiota (including opportunistic pathogenic bacteria)’ is contradicted by the increased levels of ‘potentially pathogenic’ P. distasonis mentioned in line 477, as well as other opportunistic pathogens such as Staphylococcus, Corynebacterium, and Erysipelatoclostridium, which were observed to increase in the SJBA group (Fig. 1D).

**Reply**: Thanks for pointing out this problem. Actually, what we wanted to express truly was that intervention of *B. amyloliquefaciens* could inhibit *E. coil* which was opportunistic pathogenic bacteria. Because after infection with *Schistosoma japonicum*, the abundance of *E. coli* in the gut of mice even exceeded 50% (figure below), making it become the most influential opportunistic pathogen. Therefore, we mainly focused on *E. coli* and did not pay attention to other opportunistic pathogens with lower abundance in the analysis process. It was our mistakes in writing and we have deleted this sentence.

7. Line 496: ‘the metabolites from degradation of flavonoid’ were not mentioned in the Result section. New result should not appear in the Discussion section.

**Reply**: Although this result was presented in the Figure 3D, we have added the description of this result in the results section. Please refer to line 373-374: “while degradation of flavonoids was the most significant metabolic pathways in the microbiota source”.

8. Line 499: ‘Flavonoids are polyphenolic compounds with low bioavailability, which are usually metabolized by intestinal enzymes at epithelial cells or intestinal microbiota’ is not supported by ref #77. Correct the citation.

**Reply**: We have added citation to support this sentence, please refer to line 510.

9. Line 500: 'Its metabolites have been generally shown to improve intestinal barrier function.' Reference #78 supports that 'flavonoids' improve intestinal barrier function, not 'its metabolites.'

**Reply**: We have changed it into “Flavonoids are usually metabolized by intestinal enzymes at epithelial cells or intestinal microbiota for its low bioavailability, and have been generally shown to improve intestinal barrier function and limit the exacerbation of inflammation” in line 508-510.

10. Line 505: ‘It was worth noting that the changes of 3, 4-dihydroxyhydrocinnamic acid in this process were extremely huge (fold change was close to 26), and there was a significant positive correlation with A. dispar’. The correlation between 3, 4-dihydroxyhydrocinnamic acid and A. dispar were not mentioned in the Result section. New result should not be introduced in the Discussion section. This issue arises multiple times. Please review and revise the Results and Discussion sections accordingly.

**Reply**: Thanks for pointing out this problem. Actually, we have not mentioned in the results section about this result and we have deleted it. But it was not our new result. Although we haven’t described this result, we have shown this in the Figure 4C. But we decided to deleted it in order to avoid misunderstanding. And we have checked this problem in the whole discussion to ensure to only discuss results obtained in this study.

11. Besides, what is the biological significance of these correlations to the disease? This problem also appears repeatedly, with results being repeated and some previous findings, like ‘it was related to the immune balance of Th1/2 and Treg/Th17 Cells’ in line 508’ listed in the discussion. Another example is in line 535 to 538. If they do not have a clear biological meaning or relevance to the disease/treatment, these redundant listings should be removed.

**Reply**: Thanks for pointing out this problem. We agreed with your opinion. We have deleted these descriptions.

12. Line 512: ‘genes expressing tyrosine biosynthesis’ is incorrect. It should be revised to "genes encoding enzymes involved in tyrosine biosynthesis.

**Reply**: We have changed it into “genes encoding enzymes involved in tyrosine biosynthesis” in line 519.

13. Line 535: Add citation.

**Reply**: We have added citation in line 537.

14. Line 570: How do the results from the histopathological analysis and dynamic changes in microbiota support the assertion that the intervention with B. amyloliquefaciens effectively inhibited both the pathological symptoms and the disturbance of intestinal microbiota in mice infected with S. japonicum during the acute stage?

**Reply**: Thanks for your question. However, we have deleted this sentence based on other reviewers’ comments. Based on the results of previous studies (doi: 10.3389/fcimb.2023.1172298) and current investigations, the results of histopathological analysis of the liver and intestines of model mice showed that the area of fibrosis and granuloma in the liver and intestines was significantly reduced after intervention of *B. amyloliquefaciens*. At the same time, it also reduced the mortality rate of the model mice and significantly restored their body weight. These results indicated that intervention of *B. amyloliquefaciens* effectively alleviated the pathological symptoms of the model mice. In addition, the intestinal diversity of mice was significantly restored, the community composition structure was improved, the abundance of opportunistic pathogens (*E. coil*) was greatly reduced, and the core microbiota was protected. These results revealed that intervention of *B. amyloliquefaciens* could improve disruption of gut microbiota in mice infected with *Schistosoma japonicum*. We also explained the results about how disturbance of intestinal microbiota was improved in the discussion section. Please refer to line 451-456: “Intestinal homeostasis is mainly represented by the process by which the niche of the intestinal microbiota remains stable and undisturbed [20]. Although the composition and abundance of intestinal microbiota have been fluctuating all the time due to external interventions such as diet and probiotics, the core microbiota containing key species remained stable[20]. Through the competition and cooperation within the microbiota, the healthy gut has always maintained a state of dynamic balance [68].” And line 472-474: “One of the ways that probiotics regulate intestinal homeostasis also includes improving the overall structure of intestinal microbiota by increasing the abundance of beneficial bacteria in the gut by promoting the growth of endogenous ideal microbial populations [72].”.

15. Line 602: ‘We found that the intervention of B. amyloliquefaciens could restore the dysbiosis of intestinal microbiota in infected mice, promote the growth of beneficial bacteria in the intestine, and inhibit potential pathogenic bacteria.’ It should be ‘restore the balance’.

**Reply**: Thanks for your modification. However, we have deleted this sentence based on other reviewers’ comments.

16. Fig. 5: What data was used for the ‘horizontal axis in genes proportion’? Four out of seven functional genes were not mentioned in the text to describe their relative to the intervention of B. amyloliquefaciens in mice infected with S. japonicum. Why were they chosen in the illustrated plot?

**Reply**: Thanks for pointing out this problem. The data of functional genes showed the relative proportion of one gene between two groups. The data was obtained from that the abundance of one gene divided by the total abundance which was obtained by adding up the abundance of one gene in cluster1 and cluster3 (shown below). And the relative proportion of this gene in each group could be obtained. This could more intuitively reflect the abundance of genes within two groups. And based on your suggestion, we have redrawn Figure 5. And functional genes presented in the figure were selected from Figure 4D, which was significantly enriched in cluster 1 (Figure 4A). We also have modified the category of metabolites shown in the Figure 5 based on the results of Figure 4D. Please refer to figure below and in the manuscript.

**Parts of Editorial and Data Presentation Modifications**

The manuscript is overly verbose, especially in the Discussion section. The narrative lacks clarity and logical flow. I recommend removing unnecessary and illogical statements and adding appropriate conjunctions to improve clarity and coherence. Additionally, while the study offers insights through metagenomics and metabolomics on the intervention of B. amyloliquefaciens in S. japonicum infection, supported by previous molecular findings, it remains speculative. The manuscript should avoid presenting overly definitive conclusions before further validation studies are conducted.

Besides, misleading or incorrect use of concepts can confuse readers and undermine the study's credibility.

**Reply**: Thank you for your insightful evaluation and suggestions. We fully agreed with your evaluation of our manuscript. Based on your suggestions, we have made revisions and deletions to the manuscript, especially in the discussion section. In the discussion section, we have removed some results to avoid repeated statements and reduced overly clear conclusions to minimize confusion and misleading to readers. I hope our revised manuscript satisfies you. Specific revisions were shown as following.

1. Line 59: Schistosoma japonicum is not a type of schistosomiasis, it is a species of schistosomes could lead to schistosomiasis.

**Reply**: Thanks for pointing out this problem. We have changed it into “*Schistosoma japonicum* is a parasitic worm that causes schistosomiasis” in line 59.

2. Line 64: The symptoms are primarily found in the gastrointestinal tract, liver, and spleen, with kidney involvement reported only in some cases.

**Reply**: Thanks for pointing out this problem. it was our mistakes on writing. We have changed our expression in to “Clinically, the specific symptoms are mainly concentrated in the gastrointestinal tract, liver and spleen” in line 64-65.

3. Line 74: the sentence ‘Intestinal microbiota is a complex community living in the human colon’ might suggest that the intestinal microbiota is exclusive to the colon, which can be misleading since microbiota is present throughout the gastrointestinal tract, including the small intestine.

**Reply**: Thanks for pointing out this problem. We have changed it into “Gut microbiota is a diverse community of microorganisms that reside in the gastrointestinal tract of humans and other animals” in line 74-75.

4. Line 114: Should be ‘adaptive feeding’.

**Reply**: We have changed it into “adaptive feeding” in line 125.

5. Line 174: ‘bin’.

**Reply**: It was our mistake in writing. We have deleted this sentence in line 206.

And, please pay attention to the typo, grammatical accuracy and proper formatting.

1. Line 48: the phrase 'secretion of derived metabolites derived from intestinal microbiome in mice' contains a redundancy.

**Reply**: We have changed it into “the secretion of metabolites derived from intestinal microbiome” in line 48.

2. Note the use of singular and plural forms. For example, line 284: 'indicates', 'genera'; line 378:’was’.

**Reply**: Thanks for your suggestion. We have changed it and checked these problems in the manuscript.

3. Figure legends and reference list: The name of bacteria and parasite should be italicized.

**Reply**: Thanks for your checking. We have checked this problem and modified the name of bacteria and parasite to italic.

4. Line 258: typo, ‘Erysipelatoclostridium’.

**Reply**: We have changed it into “*Erysipelatoclostridium’*” in line 294.

5. Line 293: ‘between three clusters’, should be ‘among three clusters’.

**Reply**: We have changed it into “among three clusters” in line 323.

6. Line 373: ‘related these’ should be ‘related to these’.

**Reply**: We have revised it into “related to these” in line 392.

7. Numbers at the beginning of a sentence should always be spelled out in words rather than written as Arabic numerals.

**Reply**: Thanks for your suggestion. We have changed Arabic numerals at the beginning of a sentence into spelled-out words. Please refer to line 402: “Forty-six”.

8. Line 383: ‘miniest cross validation error’ should be ‘lowest cross validation error’.

**Reply**: We have changed it into “lowest cross validation error” in line 403.

9. Line 374: ‘genes mapping of’ should be ‘genes mapping to”

**Reply**: We have changed it into “genes mapping to” in line 393.

10. Line 371: ‘multi omics’ should be revised to ‘multi-omics’.

**Reply**: We have changed it into “multi-omics” in line 389.

**Parts of Summary and General Comments**

The manuscript provides an analysis of the effects of Bacillus amyloliquefaciens on Schistosoma japonicum-infected mice using multi-omics approaches, integrating both metagenomic and metabolomic data to explore changes in the intestinal microbiome and host metabolism. Although the study offers intriguing insights into the potential benefits of B. amyloliquefaciens treatment, the manuscript requires substantial revisions to improve clarity, coherence, and accuracy. The Discussion section, in particular, is excessively detailed and lacks clarity, with many statements being speculative and not well-supported by data, which undermines the overall coherence of the manuscript. Additionally, there are discrepancies between the reported results and the conclusions drawn, especially concerning the effects of B. amyloliquefaciens on intestinal microbiota and pathology. New results are introduced in the Discussion section without prior mention in the Results section,

leading to further inconsistencies. Methodological concerns also need addressing, including the timing of B. amyloliquefaciens administration, the choice of blood collection method, adherence to ethical standards in animal research, and consistency in analysis methods.

**Reply**: Thank you for your patient feedback and detailed review comments. We fully agreed with your opinion, which will greatly help improve the quality of our manuscript. Based on your suggestions, we have made significant revisions to our manuscript. In the methods section, we have clarified the administration time of *B. amyloliquefaciens*, description of the blood collection method, consistency of the analysis method, and detailed description of the analysis method. In the results section, we reanalyzed Figure 4D and presented comparison of the results for each group, making the presentation of the results clearer and more specific. In the discussion section, we reduced the repetitive description of experimental results, modified and reduced a large number of speculative statements, and deleted some incorrect new results. We hope that our revised manuscript will meet your satisfaction.

**Response to Reviewer 4**

In this manuscript Chen et al describe the probiotic role of Bacillus amyloliquefaciens in dysregulated gut microbiota caused by Schistosoma japonicum. This investigation will provide a new strategy for adjuvant therapies of S. japonicum infection. The authors use an impressive variety of methods to test Multi-omics changes by administration of B. amyloliquefaciens and present a large amount of data that support probiotic potential of B. amyloliquefaciens. The presented data are quite standard for the field. The manuscript is very well written and, with one exception that is listed below, the conclusions are supported by the data shown. Given that Bacillus as combination therapy have already reported and the presented results are primarily bioinfomatic analyses. Despite observing some probiotic microbiota and metabolites are upregulated, lacking experimental evidence to confirm differential gut flora and metabolites caused by Bacillus amyloliquefaciens are relevant to the improvement of disease. Therefore, some experiments are advised be necessary for this paper as follow.

**Reply**: Thank you very much for your affirmation and valuable suggestions on our manuscript. Your suggestion also provided good ideas for our next experimental plan. Based on your suggestion, conducting relevant experiments could indeed enhance the credibility of our data. However, due to factors such as the equipment of the bacterial platform and the long time period for re-modeling, we were unable to further validate the relevant data. But in order to make our data more convincing, we added corresponding discussions in the discussion and supported our results with relevant literature. I hope our revised manuscript can satisfy you.

Major Issues:

1) Authours have previously published the alteration of gut microbiota in intestine of mice infected with Schistosoma japonicum after treatment with Bacillus amyloliquefaciens. What is difference compared to current investigation, data shown whether have overlapped?

**Reply**: Thanks for your insightful question. In summary, previous research was the foundation for the current investigation. Previous studies have revealed that intervention of *B. amyloliquefaciens* could alleviate symptoms of schistosomiasis japonica (granulomas and fibrosis) and elucidate the results of improving gut microbiota dysbiosis from the perspectives of diversity and taxonomic composition through histopathological analysis and 16S sequencing. The current investigation has conducted more in-depth analysis based on previous research. The current investigation mainly analyzed the interaction between functional genes of gut microbiota and serum metabolites of the host, in order to further improve the mechanism about how intervention of *B. amyloliquefaciens* improved symptoms of schistosomiasis japonica. Both the current investigation and previous studies have analyzed the dynamic changes of gut microbiota. However, the current investigation has clustered the gut microbiota of mice in different groups and time using the construction of DMM model, highlighting the importance of gut microbiota in the formation of types of communities in gut microbiota. At the same time, we highlighted the protective effect of intervention of *B. amyloliquefaciens* on the core microbiota by defining the occurrence pattern of gut microbiota. Therefore, the current investigates didn’t overlap with previous studies in terms of data shown, analysis methods, or visualization of results.

2) Data provided by authors showed that B*. amyloliquefaciens* promote the growth of beneficial bacteria and inhibit potential pathogenic bacteria. In vitro inhibition assay should be performed to explore the possibility of B*. amyloliquefaciens* directly inhibit pathogen *Escherichia Shigella*. Although B. amyloliquefaciens induced autophagy play a role in killing bacteria, secretory antimicrobials are often more efficient to kill bacteria residing in the same niche. And increased beneficial bacteria after with intervention of B. amyloliquefaciens also should be discussed about its antibacterial effect or association with decreasing pathogenic bacteria. If authors have these probiotic isolates, I encourage you perform an in vitro killing bacteria assay to confirm in vivo results.

**Reply**: Thanks for your suggestions. We believed that more killing bacteria assays were better to support our experimental results. However, due to the relocation of our lab and the long time period for rebuilding the model, it was difficult to obtain samples of mouse gut microbiota again in a short period of time. In the subsequent experiments, we will take your suggestions into consideration in the further experimental plan. And numerous studies have shown that *Bacillus* *amyloliquefaciens* could inhibit the growth of *Escherichia* *coli* through its metabolites (surfactant analogues, amylocyclines, bacteriocin like inhibitors), which also provided us with some ideas for our next experiments. Furthermore, one of the probiotic properties of *Bacillus amyloliquefaciens* was its ability to enhance the colonization ability of beneficial bacteria in the intestine, which has also been proven by relevant studies (like the adhesion ability of *L. reuteri* could be enhanced, doi: 10.1016/j.ijbiomac.2019.12.212). we have also added discussions on the antibacterial effects of beneficial bacteria and their relationship with opportunistic pathogens. Please refer to line 485-488 (“*P. distasonis* is an emerging intestinal anaerobic bacterium which could regulate the dysbiosis of gut microbiota and have strong co-excluding associations with potential pathogens like *Enterorhabdus* sp. which are frequently isolated from colitis patients”), line 496-498 (“It is worth mentioning that *L. reuteri* could produce reuterin, an antibacterial molecule that is bactericidal against a wide range of pathogenic species including enterohemorrhagic *E. coli* strains, which may play an important role in inhibiting the growth of *E. coli* in this study”).

3) Authors detected differential metabolites, whether these metabolites are responsible for limiting of pathogens (such as *Escherichia Shigella* ) in vivo. Authors should picked out several metabolites to test antibacterial ability in vitro. If it would be hard to obtain these metabolites, related antibacterial investigations should be included in discussion.

**Reply**: We believed that what you mentioned was very reasonable. However, the majority of gut bacteria were anaerobic, and our laboratory's microbial isolation platform was limited, making it impossible to isolate anaerobic bacteria. Therefore, it was difficult for us to obtain strains for co-culture of metabolites and strains. At present, our laboratory's microbial platform was being upgraded, and we will conduct validation of this experiment in the next research project. Meanwhile, we also discussed the interaction between metabolites and gut microbiota in the article based on your suggestions, which confirms our viewpoint. Please refer to line 513-515 (“flavonoids are also well known as antibacterial agents against a wide range of pathogenic microorganism and lead to promote the growth of beneficial microbiota such as *Lactobacillus* and *Bifidobacterium*”) and line 520-522 (“Supplementing DSS-model mice with tyrosol significantly reduced the abundance of *Proteobacteria* and *Shigella*, indicating the potential anti-pathogenic effect of tyrosol”).

4) I can not acquire colonization information about B*. amyloliquefaciens* from this paper, does it can colonize in intestine of mice and how long persist？If it can not survive or colonize in vivo well, whether non-living cells Bio-adjuvant would produce similar changes as observed after administration of living cells, if so, non-living cells may be safety.

**Reply**: Thank you very much for your question. We acknowledged that this was a crucial issue for our research. *Bacillus* *amyloliquefaciens* had the properties of strong acid resistance and high temperature resistance (doi: 10.1021/acs.jafc.0c06396), which allowed it to withstand the corrosion of stomach acid and reach the intestine. Secondly, it had been proven that *Bacillus* *amyloliquefaciens* could colonize in the intestinal tract of dogs (doi: 10.1016/j.micpath.2017.11.040), indicating its intestinal adhesion and ability to colonize and regulate gut microbiota in animals’ intestines. The results of these studies led us to believe that *Bacillus* *amyloliquefaciens* had the ability to reach the animals’ gut and exert probiotic effects. But as you said, if we could directly provide data on colonization of *Bacillus* *amyloliquefaciens*, it would make our research more convincing. We have also taken this into consideration, so our future research will definitely conduct this experiment. Furthermore, inspired by your comment, we will take your suggestion into full consideration when considering the form of the Bio-adjuvant after determining the effectiveness of our probiotic. Thank you very much.

5) IFN-γ, IL-12, TNF-α, and IL-4, IL-5, IL-13, TGF-β were shown to increase during infection, authors should measure these cytokines whether have changes after administration of Bacillus amyloliquefaciens, which should be considered for its application value in clinical treatment.

**Reply**: Thank you for your suggestion. We fully agreed with your opinion. However, due to the long time period for conducting another experiment and the need to re-feed the cercaria. Therefore, we considered measuring the indicators of inflammatory factors in the subsequent experimental plan.

Minor Issues:

I suggest following changes in writing:

Line 47 dysbiosis should be replaced by restoration.

**Reply**: We have changed “dybiosis” into “restoration” in line 47.

Line 102 “which can target and reshape the intestinal microbiota and has the potential to alleviate schistosomiasis japonica” should be edited as “which has the potential to alleviate schistosomiasis japonica by targeting and reshaping the intestinal microbiota” because previous sentence have mentioned the role of *B. amyloliquefaciensa* regulating gut microbiota.

**Reply**: We have changed it into “which has the potential to alleviate schistosomiasis japonica by targeting and reshaping the intestinal microbiota” in line 102-103.
